# Mitochondria‐Targeted Multimodal Nanotherapeutics Suppress Oxidized mtDNA‐Driven Inflammation at the Source

**DOI:** 10.1002/advs.77577

**Published:** 2026-09-01

**Authors:** Wen‐Ling Li, Juan Cao, Yu‐Lin Wang, Yu‐Han Fan, Jia‐Qi Xu, Xian Wu Cheng, Lei Xing, Hu‐Lin Jiang

**Affiliations:** ^1^ State Key Laboratory of Natural Medicines Department of Pharmaceutics China Pharmaceutical University Nanjing China; ^2^ Jilin Provincial Key Laboratory of Stress and Cardiovascular Disease Department of Cardiology and Hypertension The Affiliated Hospital of Yanbian University (Yanbian University Hospital) Yanji China; ^3^ Joint International Research Laboratory of Target Discovery and New Drug Innovation MOE China Pharmaceutical University Nanjing China; ^4^ Department of Precision Medicine School of Medicine Sungkyunkwan University Suwon South Korea

**Keywords:** acute respiratory distress syndrome, flap endonuclease 1, inflammation, mitochondrial targeting, oxidized mitochondrial DNA

## Abstract

Oxidized mitochondrial DNA (Ox‐mtDNA) fragments escaping from mitochondria trigger a potent inflammatory cascade that endangers cellular and tissue integrity. Strategies against pre‐existing mtDNA‐driven inflammation have been developed. However, the inhibition of emerging Ox‐mtDNA fragments remains a critical challenge due to continual leakage from stressed mitochondria. Here, we present a source‐control nanodelivery strategy that targets Ox‐mtDNA to prevent the generation of fragments and eliminate existing ones. To this end, a small molecule inhibitor FI is specifically designed to selectively target mitochondrial flap endonuclease 1 (mitochondrial FEN1), thereby inhibiting Ox‐mtDNA cleavage and the subsequent leakage of fragments. To facilitate the precise and efficient delivery of FI to inflammation‐associated cells, we engineered a hyaluronic acid (HA) functionalized nanocarrier, designated Se‐FI@Lip‐HA, which encapsulates selenium nanoparticles bearing FI (Se‐FI NPs) within phospholipid vesicles. The SeNPs further scavenge reactive oxygen species (ROS) and promote autophagy‐mediated clearance of leaked mtDNA. Acute respiratory distress syndrome (ARDS), as a severe inflammatory lung disorder, is used as a proof‐of‐concept model. Se‐FI@Lip‐HA multimodally suppresses inflammatory signaling activated by Ox‐mtDNA at the source and effectively alleviates lung tissue injury. This strategy offers a broadly applicable therapeutic avenue for Ox‐mtDNA‐driven pathologies in ARDS and other inflammatory diseases.

## Graphical abstract

Ox‐mtDNA fragments escaping from mitochondria drive robust inflammatory responses. A targeted nanoplatform simultaneously inhibits mitochondrial FEN1‐mediated mtDNA cleavage and scavenges ROS, preventing the generation of immunogenic Ox‐mtDNA fragments. The released SeNPs further promote autophagic clearance of cytosolic mtDNA. Consequently, cGAS‐STING, NLRP3, and TLR9 inflammatory cascades are suppressed at the source.

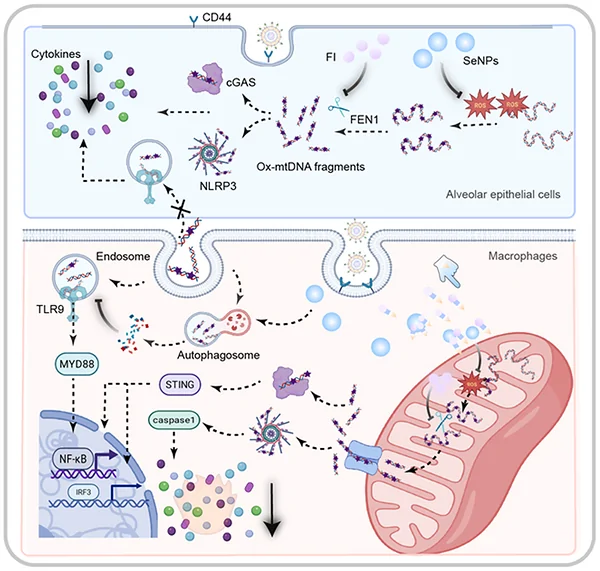

## 1 Introduction

As an endogenous red flag, the critical role of oxidized mitochondrial DNA (Ox‐mtDNA) in inflammation is increasingly emphasized [1, 2, 3]. Recent studies have shown that numerous inflammatory disorders, such as autoimmune diseases, cardiovascular diseases, neurodegenerative diseases, and acute severe infections, have been linked to Ox‐mtDNA [4, 5, 6, 7, 8]. Many approaches have been proposed to suppress mtDNA‐activated inflammatory pathways, such as using small molecule inhibitors or antagonists of cGAS, NLRP3, or TLR9 [9, 10, 11]. Although these strategies hold promise for suppressing inflammatory responses, the presence of cytoplasmic mtDNA leads to a persistent inflammatory response. In this regard, mtDNA clearance strategies (e.g., DNases or cationic polymers) have been introduced [12, 13]. Nevertheless, the continued leakage of Ox‐mtDNA with higher immunogenicity from stressed mitochondria is overlooked in these studies.

Mitochondria play an essential role in inflammation and reactive oxygen species (ROS) generation [14, 15]. Under mitochondrial stress, mtDNA replication can be enhanced. This newly synthesized mtDNA is particularly sensitive to ROS, and oxidative damage promotes its release into the cytoplasm [16]. Because of its bacterial ancestry and CpG‐rich features, mtDNA can act as a potent endogenous agonist of innate immune signaling when exposed to the cytosol or extracellular space [17]. It is worth noting that the liberation of mtDNA from mitochondria into the cytosol requires its prior fragmentation. Previous studies suggest that cleavage into fragments below approximately 700 bp facilitates mtDNA passage through mitochondrial membrane pores. This cleavage is facilitated by mitochondrial flap endonuclease 1 (mitochondrial FEN1), a pivotal enzyme in nucleic acid cleavage [18]. Previous studies have shown that cytosolic mtDNA is frequently oxidized and fragmented across different cell types [19]. After Ox‐mtDNA fragments enter the cytosol, they activate downstream sensors cGAS and NLRP3 inflammasome, and subsequently produce a series of inflammatory factors, including type I interferons, TNF‐α, IL‐6, IL‐1β, IL‐18, etc [20, 21]. Moreover, extracellular mtDNA endocytosed by cells forms an endosome, which activates TLR9 and further induces the release of inflammatory cytokines [22]. Altogether, the leaked Ox‐mtDNA fragments exert broader pro‐inflammatory effects, fanning the fires of inflammatory eruption within cells. Therefore, developing a strategy that integrates mitochondrial FEN1 inhibition with ROS scavenging to address the root cause of inflammation holds significant promise for treating inflammatory diseases.

As an efficient ROS scavenger, selenium nanoparticles (SeNPs) mimic the activity of glutathione peroxidase (GPX), protecting cells from oxidative damage [23]. Simultaneously, they activate autophagic pathways promoting the clearance of damaged mitochondria and leaked mtDNA [24]. This dual action makes SeNPs a promising multimodal agent for treating Ox‐mtDNA‐driven inflammatory diseases. The oxidation, fragmentation, and cytosolic release of mtDNA under mitochondrial stress initiate a potent inflammatory cascade. FEN1, as a DNA repair enzyme, is abnormally elevated under inflammatory stress, which plays a crucial role in shearing mtDNA into fragments [25]. However, the inhibitory strategies against FEN1 are currently mainly employed in tumor treatment by comprehensively interfering with DNA replication and repair [26, 27, 28]. The inherent cytotoxicity of current FEN1 inhibitors prevents their therapeutic application in inflammatory diseases. How to effectively and safely avoid the formation of Ox‐mtDNA fragments at the source remains a critical concern for achieving inflammatory treatment.

**FIGURE 1:**
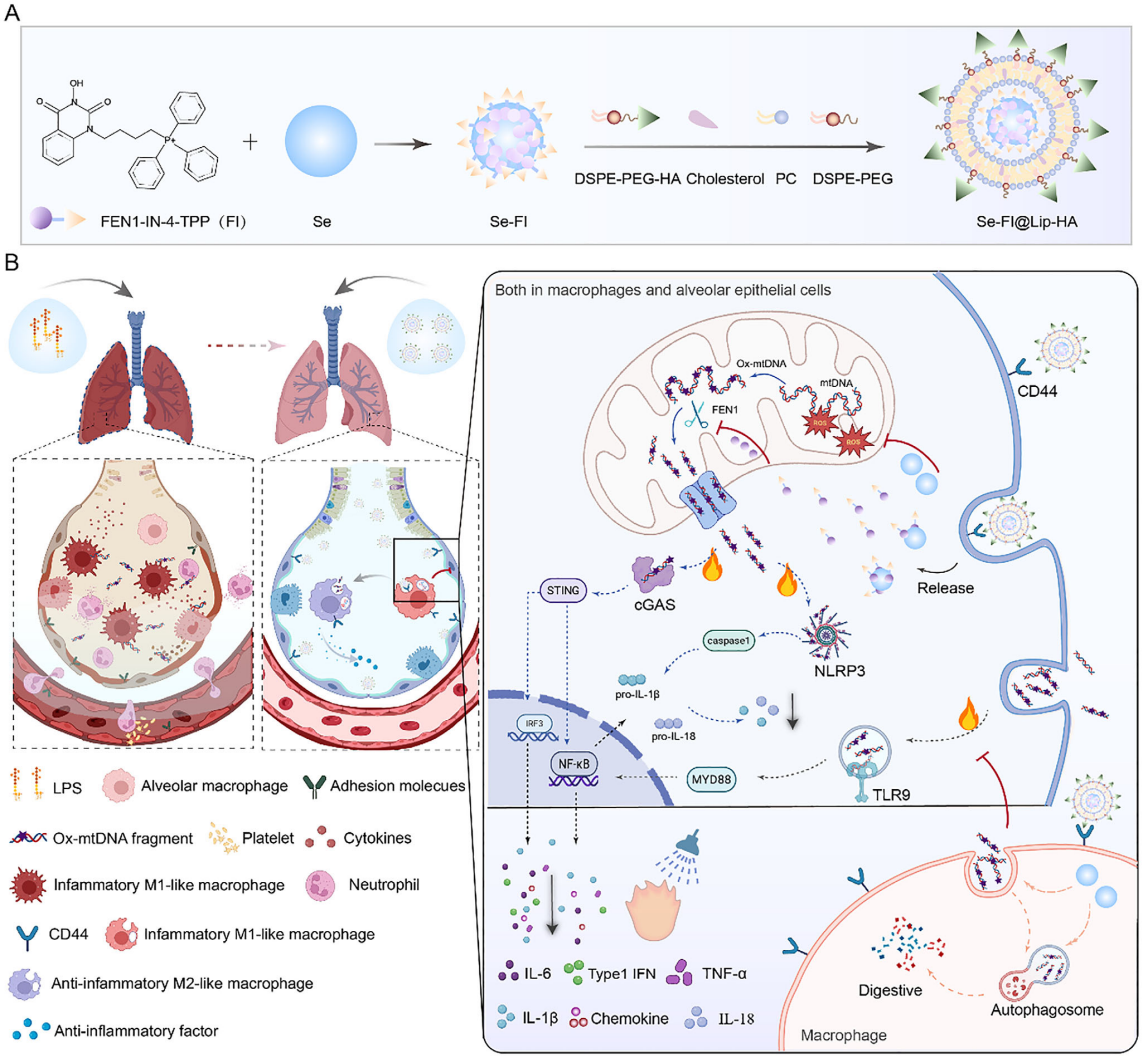
Schematic illustration of Se‐FI@Lip‐HA for the treatment of ARDS. (A) The construction of Se‐FI@Lip‐HA. (B) Schematic representation of Se‐FI@Lip‐HA for ARDS treatment and mechanism representation of multimodally blocking the flames of inflammation ignited by Ox‐mtDNA fragments. Se‐FI@Lip‐HA inhibits the production of Ox‐mtDNA fragments both in macrophages and alveolar epithelial cells, as well as promotes macrophage endocytosis and autophagy to clear extracellular mtDNA.

Here, we constructed a small‐molecule FI that selectively targets mitochondrial FEN1 by linking TPP, a mitochondrial target to FEN1‐IN‐4, a classical FEN1 inhibitor. FI preferentially accumulates in mitochondria and inhibits mitochondria‐associated FEN1 activity, which curtails the generation of mtDNA fragments while mitigating its potential to disrupt nuclear homeostasis and incite inflammation. To achieve targeted delivery of FI to inflammatory cells, selenium nanoparticles bearing FI (Se‐FI NPs) were encapsulated by hyaluronic acid (HA) functionalized phospholipid vesicles for the construction of an anti‐inflammatory nanoparticle (Se‐FI@Lip‐HA). After uptake by inflammatory cells, Se‐FI NPs localize to the mitochondrial vicinity, where they scavenge ROS, a major contributor to mtDNA oxidation, thereby reducing the level of Ox‐mtDNA. Se‐FI NPs not only inhibit oxidative damage to mtDNA, but also block the cleavage of mtDNA to avoid the leakage of fragments. Moreover, enhanced phagocytosis and autophagy promote clearance of extracellular mtDNA by macrophages. Acute respiratory distress syndrome (ARDS) is a life‐threatening inflammatory lung disorder characterized by diffuse alveolar damage and dysregulated production of pro‐inflammatory mediators, which lead to lung injury and respiratory failure and high mortality [29, 30]. Despite improvements in clinical management, current treatment remains largely supportive care, while effective pharmacological therapies are still limited [31]. To validate the therapeutic efficacy of Se‐FI@Lip‐HA in inflammatory diseases, we introduce ARDS as a proof‐of‐concept model (Figure 1A,B). Cellular and murine data demonstrate that Se‐FI@Lip‐HA is efficiently taken up by macrophages and epithelial cells, leading to a marked reduction in Ox‐mtDNA levels and attenuation of downstream inflammatory signaling. In ARDS mice, treatment with Se‐FI@Lip‐HA significantly decreased the levels of cell‐free mtDNA (cf‐mtDNA) and pro‐inflammatory factors in bronchoalveolar lavage fluid (BALF). Moreover, Se‐FI@Lip‐HA robustly inhibited the generation of Ox‐mtDNA fragments and alleviated acute lung injury in a sepsis model, thereby extending its potential applicability to other inflammatory diseases. Overall, by integrating mitochondrial FEN1 inhibition with ROS scavenging, this strategy blocks Ox‐mtDNA‐mediated inflammatory amplification at the source and provides a broadly applicable therapy for inflammatory diseases marked by persistent Ox‐mtDNA release.

## 2 Results and Discussion

### 2.1 Elevated Endonuclease Expression, Cytosolic DNA‐Mediated Inflammation, and Redox Imbalance in ARDS

**FIGURE 2:**
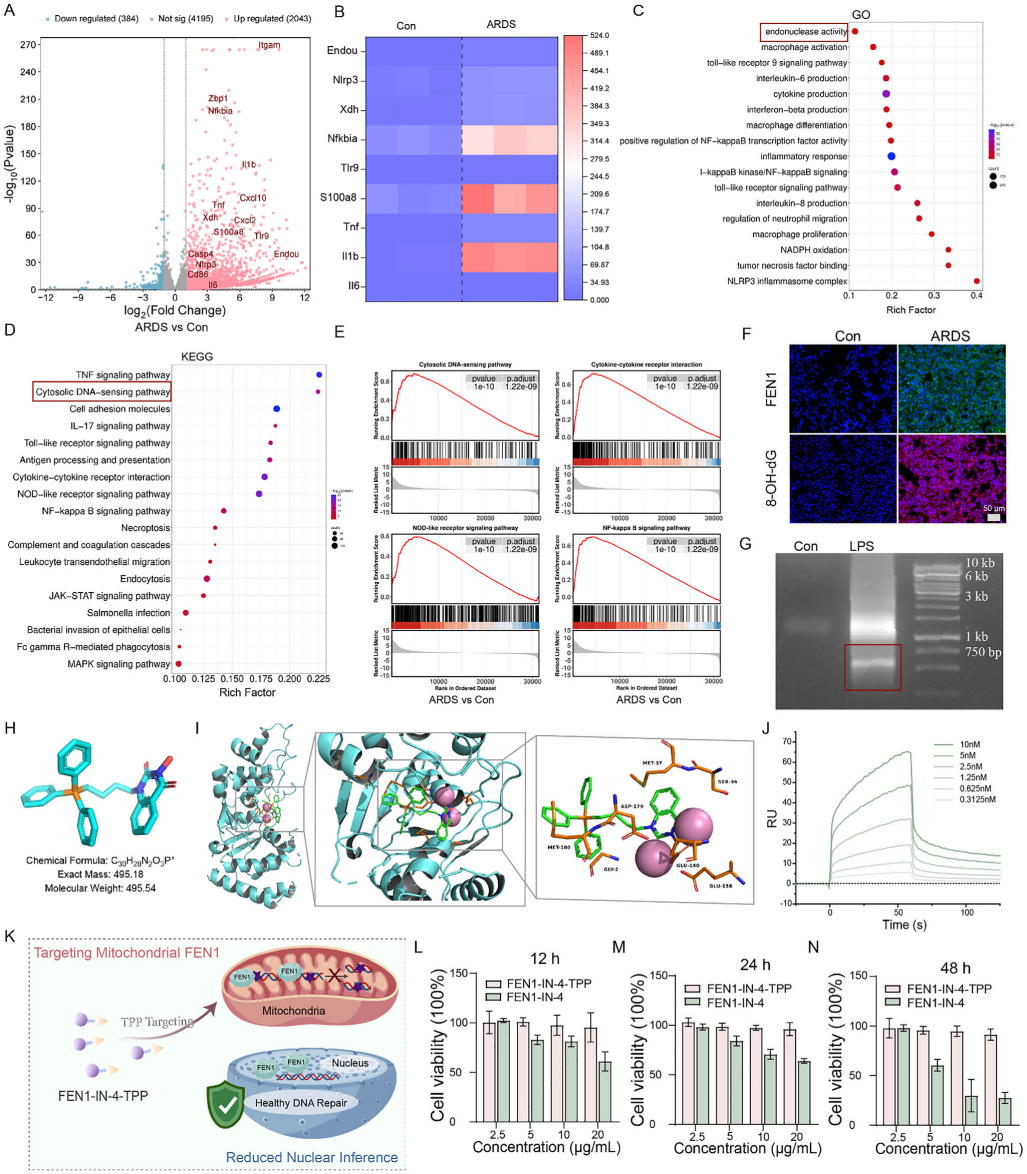
Key features of ARDS and FI synthesis. (A) Genome‐wide analysis. The volcano plot showing differentially expressed genes between the control (Con) and ARDS groups in the lung tissue of mice (*n* = 3). (B) Heatmap analysis of endonucleases and inflammatory pathway genes corresponding to A (*n* = 3). (C) GO analysis based on the significantly differentially expressed genes between Con and ARDS. (D) KEGG enrichment analysis. The most enriched pathways are downstream inflammatory pathways activated by mtDNA. (E) Representative GSEA plots based on KEGG. (F) Immunofluorescence images of lungs in the Con and ARDS groups. Scale bar: 50 µm. (G) Cytosolic mtDNA was detected and separated by nucleic acid gel electrophoresis. (H) Molecular structural formula of FI. (I) 3D structure of FI in FEN1 protein (pink spheres represent magnesium ions). (J) Interaction affinity of FEN1 protein and FI measured by SPR. (K) Schematic illustration of the mechanism underlying FI‐enhanced biosafety. (L–N) Cell viability of MLE‐12 incubated with FI and FEN1‐IN‐4 for different times, respectively (*n* = 6). Data are presented as mean ± SD (L, M, N).

Inflammation is a common pathological process, whereas extreme inflammation may be fatal [32, 33]. Notably, ARDS is an inflammatory lung syndrome with significant mortality rates as high as 30% to 40% [30, 34]. To explore the role of mtDNA in igniting inflammation, the mouse model of ARDS induced by intratracheal instillation of lipopolysaccharide (LPS) was used. We performed RNA‐seq on the lungs of the control group and the ARDS group. The results showed that after 24 h of LPS induction, the expression of endonuclease‐related, oxidative stress‐related, and inflammatory genes was upregulated in ARDS compared to the control group (Figure 2A,B).

Gene Ontology (GO) and Kyoto Encyclopedia of Genes and Genomes (KEGG) analysis showed that genes were enriched in endonucleases, ROS‐producing enzyme systems, and downstream inflammatory pathways activated by cytoplasmic DNA (Figure 2C,D). We also analyzed the cytosolic DNA‐sensing pathway, LPS recognition pathway as well as the inflammatory signaling pathway by Gene Set Enrichment Analysis (GSEA). The results indicated that these pathways are upregulated in ARDS (Figure 2E and Figure S1). As a vital DNA endonuclease, mitochondrial FEN1‐generated Ox‐mtDNA fragments exit mitochondria activating inflammation. Immunofluorescence of FEN1 and 8‐Hydroxy‐2'‐deoxyguanosine (8‐OH‐dG) a marker of DNA oxidative damage was investigated, which indicated that ARDS undergoes high FEN1 expression and 8‐OH‐dG production (Figure 2F). Subsequently, the size of mtDNA in the cytoplasm was analyzed by agarose gel electrophoresis, and the results showed that the DNA fragments less than 750 bp in the cytoplasm of the ARDS group were significantly increased compared to the control group (Figure 2G). These findings suggested that the enhanced expression of FEN1 promotes the activation of inflammatory pathways caused by Ox‐mtDNA. FEN1 inhibitors are mainly used in tumor treatment at present, which lack specific mitochondrial targeting, limiting their broader application in inflammatory diseases [26, 27]. Therefore, we have developed a mitochondria‐targeted FEN1 inhibitor and the FI was obtained in only a two‐step reaction (Figure S2A). As shown in Figure 2H, the 3D structure of FI, where TPP acts as a mitochondrial target, is linked to FEN1‐IN‐4, an effective FEN1 inhibitor. Then, FI was characterized by liquid chromatography‐mass spectrometry and ^1^H nuclear magnetic resonance spectroscopy (^1^H NMR), and the final purity was 95% (Figure S2B,C).

Next, we looked into how FI affected mitochondrial FEN1. The findings indicated that they are located in the nuclease active region of the protein, where the N‐hydroxyurea moiety directly coordinates two Mg^2+^ ions, perhaps preventing access of the DNA to catalytic metal ions in FEN1 (Figure 2I and Figure S2D). In addition, we probed the interaction of FI with the FEN1 protein using surface plasmon resonance (SPR) and isothermal titration calorimetry (ITC). As shown in Figure 2J and Figure S3, FI not only has a strong binding capacity but also binds to FEN1 rapidly. By enhancing mitochondrial targeting, TPP modification was expected to reduce nuclear exposure by promoting mitochondrial accumulation, thereby improving the apparent safety profile (Figure 2K). Subsequently, we performed an MTT assay to investigate the cellular viability after incubation with FI or FEN1‐IN‐4 on murine lung epithelial‐12 (MLE‐12). It can be found that FI had no obvious toxicity, but FEN1‐IN‐4 showed increased cytotoxicity with time (Figure 2L–N). This could be the widespread inhibition of DNA in cells by FEN1‐IN‐4, which interferes with DNA replication and repair, inducing DNA damage and apoptosis. Live/dead staining further demonstrates that TPP modification reduced FEN1‐IN‐4‐associated cytotoxicity (Figure S4). These results suggest that FI has a better safety profile than FEN1‐IN‐4 while retaining comparable affinity for FEN1, making it more suitable for ARDS treatment in subsequent research applications.

### 2.2 Preparation and Characterization of Se‐FI@Lip‐HA

**FIGURE 3:**
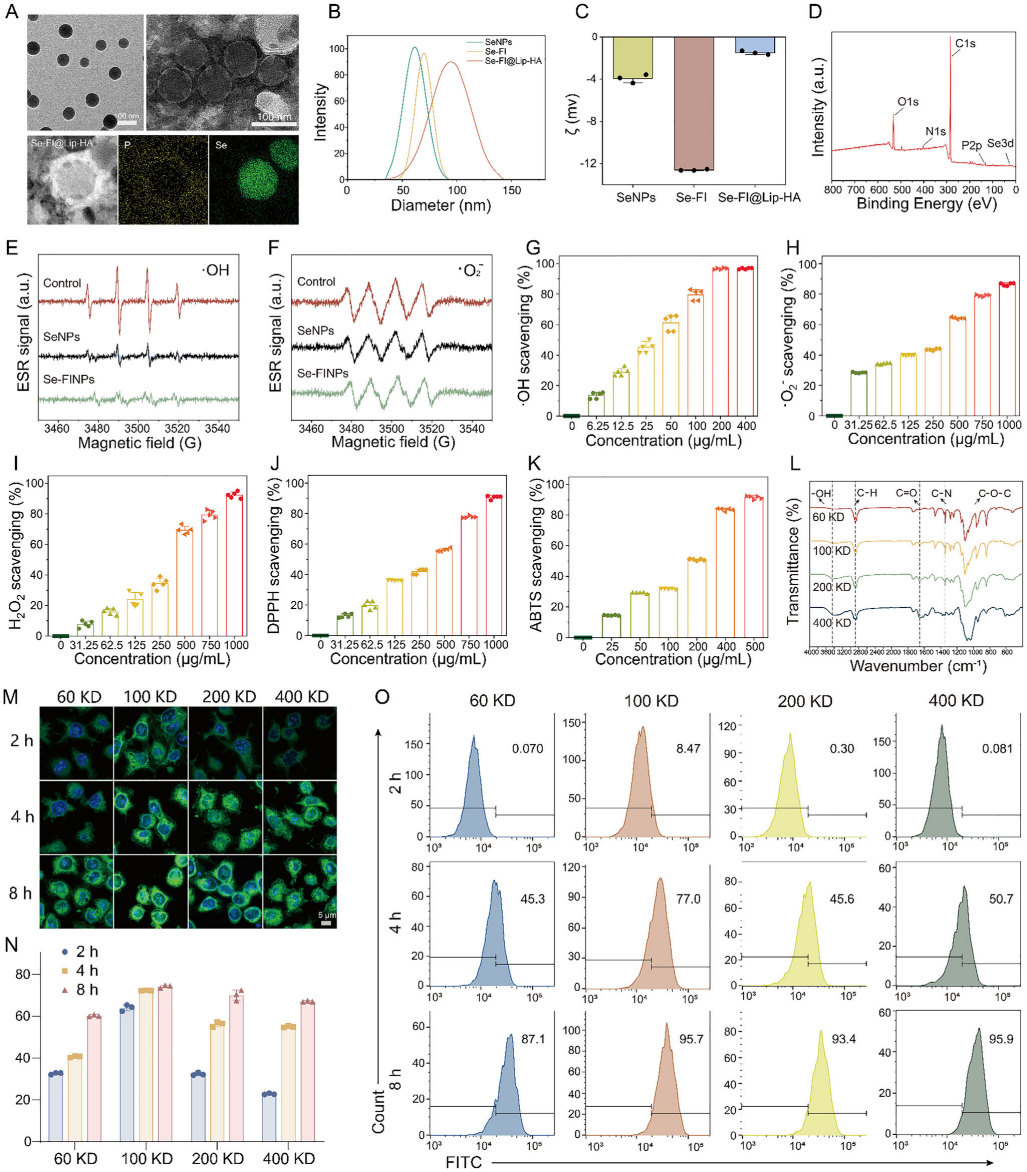
Prescription screening and characterization of nanoparticles. (A) TEM images of Se‐FI NPs and Se‐FI@Lip‐HA, and elemental mapping of Se‐FI@Lip‐HA. Scale bar: 100 nm. (B) Size distributions of various formulations. (C) Zeta potential of different formulations (*n* = 3). (D) XPS spectra of Se‐FI@Lip‐HA. (E, F) Representative ESR spectra of ·O_2_^−^ and ·OH scavenging by SeNPs and Se‐FI NPs. (G–K) Scavenging rates of SeNPs at different concentrations on ·OH, ·O_2_^−^, H_2_O_2_, DPPH, and ABTS radicals (*n* = 5). (L) FTIR spectra of DSPE‐PEG‐HA conjugates with different molecular weights of HA (MW: 60, 100, 200, and 400 KD). (M) Confocal microscopy images of RAW264.7 cell uptake of Se‐FI@Lip‐HA grafted with HA of different molecular weights at various time points (2, 4, and 8 h). (N) Quantitative analysis of fluorescence intensity from CLSM images of cellular uptake. (O) Flow cytometry analysis of cellular uptake of Se‐FI@Lip‐HA grafted with HA of different molecular weights (*n* = 3). Data are presented as mean ± SD (C, G‐K, N).

The Se‐FI@Lip‐HA was constructed by encapsulating Se‐FI with phospholipid containing HA. Uniformly sized SeNPs were synthesized using vitamin C (V_C_) by reducing Na_2_SeO_3_, in which chitosan (Cs) acted as a stabilizer [35, 36]. During this process, the optimal molecular weight of Cs and stirring time were investigated by particle size of SeNPs (Figure S5A,B). The Se‐FI NPs were prepared by physical adsorption between SeNPs and FI (Figure S5C). The drug loading (DL%) of FI was determined to be 8.30% ± 0.05%, and the cumulative release of FI from the formulation reached approximately 88.3% (Figure S6). Transmission electron microscopy (TEM) images revealed that the resultant Se‐FI NPs and Se‐FI@Lip‐HA possess a spherical shape with an average distribution of around 65.96 ± 8.141 nm and 80.02 ± 6.190 nm, respectively (Figure 3A). Meanwhile, TEM and EDS mapping were conducted to reveal the chemical composition across the Se‐FI@Lip‐HA particles, in which the expected elements, including phosphorus and selenium, were identified and matched well with the counterpart positions in the nanostructure (Figure 3A). Additionally, the successful synthesis in each stage is indicated by the dynamic light scattering (DLS) and ζ‐potential changes between Se, Se‐FI, and Se‐FI@Lip‐HA (Figure 3B,C). The peaks of C1s, O1s, N1s and P2p in the XPS spectrum of Se‐FI@Lip‐HA further demonstrated the successful incorporation of FI and phospholipid structures (Figure 3D). Subsequently, the stability of Se‐FI@Lip‐HA was examined and the result showed that the size and polydispersity index (PDI) did not significantly change in PBS, saline or 10% FBS for 7 days (Figure S7A–C).

Next, we verified the ROS scavenging ability of SeNPs. As the two main forms of ROS in biological environments, hydroxyl radicals (·OH) and superoxide anions (·O_2_ **^−^**) were examined by electron spin resonance (ESR). The results demonstrated that SeNPs greatly decreased the spectrum signals of ·OH and marginally decreased the magnitude of the ·O_2_^−^ characteristic peak (Figure 3E,F and Figure S8). Notably, the catalytic activities of SeNPs were preserved even after FI was adsorbed. Subsequently, we evaluated the capacity of various concentrations of SeNPs to eliminate ROS. Similar results confirmed that SeNPs has the activity of scavenging different types of ROS, and increasing the concentration of SeNPs will considerably boost its scavenging rates (Figure 3G–I and Figure S8). Moreover, the antioxidant potential and the total antioxidant capacity were further monitored using DPPH and ABTS, respectively. As shown in Figure 3J,K and Figure S8, the SeNPs display improved antioxidant activity with increased concentration. Compared with traditional antioxidants Vc and vitamin E (Ve) [37, 38], SeNPs showed antioxidant performance comparable to Vc and higher than Ve, laying the foundation for subsequent ROS scavenging in vivo and in vitro (Figure S9A–D).

CD44 expression in activated macrophages, especially in the pro‐inflammatory M1 phenotype, is significantly upregulated [39]. As a typical ligand to CD44, HA is modified to the surface of liposomes [40]. To investigate targeting to macrophages, we synthesized DSPE‐PEG‐HA containing different molecular weights of HA (Figure 3L). Then, fluorescent molecule Ce6‐loaded liposomes (Ce6‐Lip‐HA) containing varying molecular weights of HA were prepared based on the Se‐FI@Lip‐HA preparation process to investigate the uptake of mouse mononuclear macrophage leukaemia (RAW264.7). After confirming that the various Ce6‐Lip‐HA groups mentioned above had comparable Ce6 encapsulation rates, we conducted the subsequent cell uptake‐related studies (Figure S10). We used Ce6 and DAPI fluorescence probes to identify the Ce6‐Lip‐HA and nucleus in M1 treated with various Ce6‐Lip‐HA for different times. The results of fluorescence images demonstrated that the Ce6‐Lip‐HA cellular uptake process is time‐dependent (Figure 3M,N). More importantly, LPS‐primed RAW264.7 cells treated with the liposomal group containing 100 KD of HA showed the strongest green fluorescence, indicating that this group possesses excellent targeting, which is consistent with the flow cytometry data (Figure 3O). Based on these findings, the following cell and animal experiments were carried out using Se‐FI@Lip‐HA with 100 KD of HA.

### 2.3 Se‐FI@Lip‐HA Mitigates ROS and Reduces Ox‐mtDNA In Vitro

**FIGURE 4:**
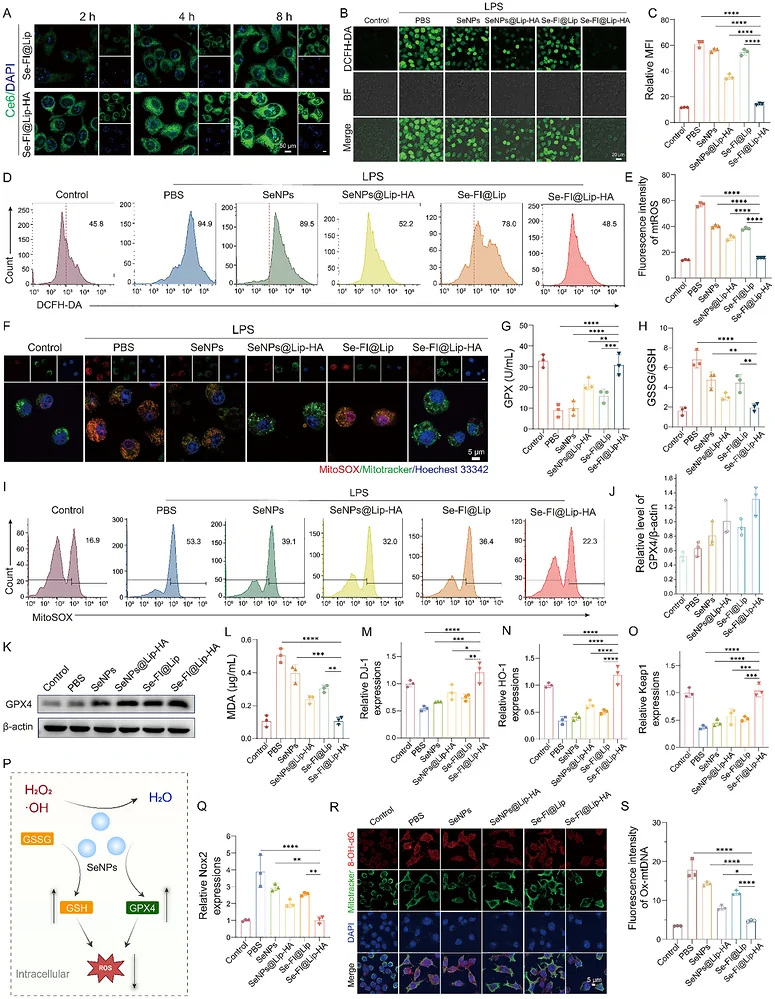
Excellent antioxidant properties of nanoparticles. (A) CLSM images showing the uptake of formulations with or without HA grafting by LPS‐primed RAW264.7 cells at different time points. Scale bar: 50 µm. (B) CLSM observation of ROS scavenging effects of SeNPs, SeNPs@Lip‐HA, Se‐FI@Lip, and Se‐FI@Lip‐HA in LPS‐induced inflammatory macrophages using DCFH‐DA probe (green). Scale bar: 20 µm. (C) Quantification of DCFH‐DA fluorescence intensity from CLSM images. (D) Flow cytometry analysis of intracellular ROS levels in inflammatory macrophages treated with different formulations. (E) Quantification of MitoSOX fluorescence intensity from CLSM images. (F) CLSM images of mtROS in RAW264.7 cells after inflammatory induction and treated with different groups. mtROS were labeled with MitoSOX (red), and mitochondria were labeled with MitoTracker Green (green). Scale bar: 5 µm. (G, H) Determination of GPX activity and GSSG/GSH ratio of inflammatory macrophages treated with different NPs (*n* = 3). (I) Flow cytometry analysis of intracellular mtROS levels in inflammatory RAW264.7 cells under different treatment groups. (J) Quantification of GPX4 protein expression levels normalized to β‐actin. (K) Western blot (WB) analysis of GPX4 protein expression in RAW264.7 cells treated with different NPs. (L) Determination of MDA levels of inflammatory macrophages treated with different NPs (*n* = 3). (M–O) RT‐qPCR analysis of DJ‐1, HO‐1, and Keap1 expression in RAW264.7 cells treated with different NPs (*n* = 3). (P) Schematic illustration of the cellular uptake of Se‐FI@Lip‐HA nanoparticles and scavenging the overproduced ROS in RAW264.7. (Q) RT‐qPCR analysis of Nox2 gene expression in RAW264.7 after different treatments. (R) Confocal immunofluorescence images of Ox‐mtDNA in RAW264.7 cells treated with different groups. Ox‐mtDNA was immunolabeled with 8‐OH‐dG rabbit antibody followed by ABflo 647‐conjugated goat anti‐rabbit IgG (red). Mitochondria were labeled with MitoTracker Green (green). Scale bar: 5 µm. (S) Quantification of 8‐OH‐dG fluorescence intensity from CLSM images. Data are presented as mean ± SD and analyzed by one‐way ANOVA with Tukey's multiple comparisons test. *p < 0.05, **p < 0.01, and ***p < 0.001, ****p < 0.0001.

Biocompatibility is essential for inflammatory treatment and future applications in vivo. To investigate the toxicity, we used the MTT assay and live/dead staining to examine different cellular vitality incubated with Se‐FI@Lip‐HA (Figure S11). The cell survival rate remained above 89.36% even after co‐incubation with Se‐FI@Lip‐HA for 48 h, indicating good cytocompatibility of the nanoplatform. Afterward, the targeting of Se‐FI@Lip‐HA to macrophages was analyzed, and we synthesized Ce6‐Se‐FI@Lip as a control group. Ce6‐Se‐FI@Lip‐HA exhibited higher fluorescence intensity at different time points owing to the binding of HA to CD44 on the surface of macrophages (Figure 4A and Figure S12). Moreover, we assessed Se‐FI@Lip‐HA's potential to protect epithelial cell viability in vitro through a co‐culture system in which murine lung epithelial‐12 (MLE‐12) cells were co‐cultured with inflammatory macrophages pretreated with different kinds of NPs. The results of live/dead staining demonstrated that both SeNPs@Lip‐HA and Se‐FI@Lip‐HA significantly increased the viability of MLE‐12 compared to the PBS group (Figure S13), which may be ascribed to their targeting and ability to scavenge ROS.

In response to LPS stimulation, excessive ROS were produced in RAW264.7 cells, causing oxidative damage [41]. Accordingly, we further assessed the intracellular ROS level using 2’,7’‐dichlorofluorescein diacetate (DCFH‐DA) probe [42, 43]. As shown in Figure 4B,C, the green fluorescence intensity associated with ROS was significantly weakened in Se‐FI@Lip and Se‐FI@Lip‐HA groups compared with other groups. The ROS level in the Se‐FI@Lip‐HA group was lower than Se‐FI@Lip group. This may be attributed to the inhibition of inflammatory pathways reducing the production of ROS [44]. Flow cytometry data further supported Se‐FI@Lip‐HA's efficient ROS scavenging properties (Figure 4D). Additionally, we evaluated the capacity of various concentrations of Se‐FI@Lip‐HA to eliminate ROS. As shown in Figure S14, the fluorescence intensity of ROS in cells was markedly attenuated after co‐incubation with Se‐FI@Lip‐HA, and there was a positive correlation between the attenuation of fluorescence and Se‐FI@Lip‐HA concentration. Simultaneously, the MitoSOX probe was employed to assess mitochondrial ROS levels [45]. The MitoSOX intensity in the Se‐FI@Lip‐HA group was lower than that of other groups, suggesting that Se‐FI enhanced the scavenging of ROS around mitochondria (Figure 4E,F,I and Figure S15).

To study the targeting of Se‐FI to mitochondria, we further detected mitochondrial Se content in macrophages after treatment with Se‐FI@Lip‐HA or SeNPs@Lip‐HA. As shown in Figure S16, Se‐FI@Lip‐HA treatment significantly increased mitochondrial Se content, which was consistent with MitoSOX results.

The down‐regulation of ROS levels in inflammatory regions is crucial for mitigating inflammation and preventing tissues from oxidative damage [46, 47]. GPX and glutathione (GSH) are the key substances for maintaining redox balance [48, 49]; we further observed their changes in cells after different drug treatments. The level of GPX was markedly enhanced in RAW264.7 cells in the Se‐FI@Lip‐HA group compared to other groups (Figure 4G). This may be attributed to the fact that many GPX family enzymes, including GPX4, are selenoproteins [50], and supplementation with Se NPs increases GPX expression (Figure 4J,K). Additionally, the overall oxidative stress index in cells, GSSG/GSH, was significantly down‐regulated in the Se‐FI@Lip‐HA group (Figure 4H). As a result, the degree of lipid peroxidation damage of the cell membrane after Se‐FI@Lip‐HA treatment was significantly improved (Figure 4L). These findings indicated that Se‐FI@Lip‐HA was effective in upregulating GPX and downregulating GSSG/GSH and MDA, which was positively associated with its concentration (Figure S17). Subsequently, quantitative reverse transcription PCR (RT‐qPCR) analysis was used to measure the levels of redox‐related genes. The redox‐regulatory genes, including DJ‐1, HO‐1, and Keap1 in the Se‐FI@Lip‐HA group were increased, whereas the expression of the pro‐oxidant gene Nox2 was down‐regulated (Figure 4M–O, Q). As summarized schematically in Figure 4P, these results confirmed the effective ROS‐scavenging capability of Se‐FI@Lip‐HA in vitro. Based on the above results, we analyzed the levels of Ox‐mtDNA in cells after different treatments. 8‐OH‐dG is an oxidative adduct produced by ROS attacking guanine bases in DNA molecules, and is widely used as a biomarker of DNA oxidative damage [51]. Therefore, we analyzed the level of 8‐OH‐dG by immunofluorescence staining, and the results showed that the Ox‐mtDNA was significantly reduced in the Se‐FI@Lip‐HA group (Figure 4R,S). These results suggested that Se‐FI@Lip‐HA therapy can mitigate ROS and decrease Ox‐mtDNA in mitochondria.

### 2.4 Se‐FI@Lip‐HA Reduces the Amount of Cytoplasmic and Extracellular mtDNA Fragments

**FIGURE 5:**
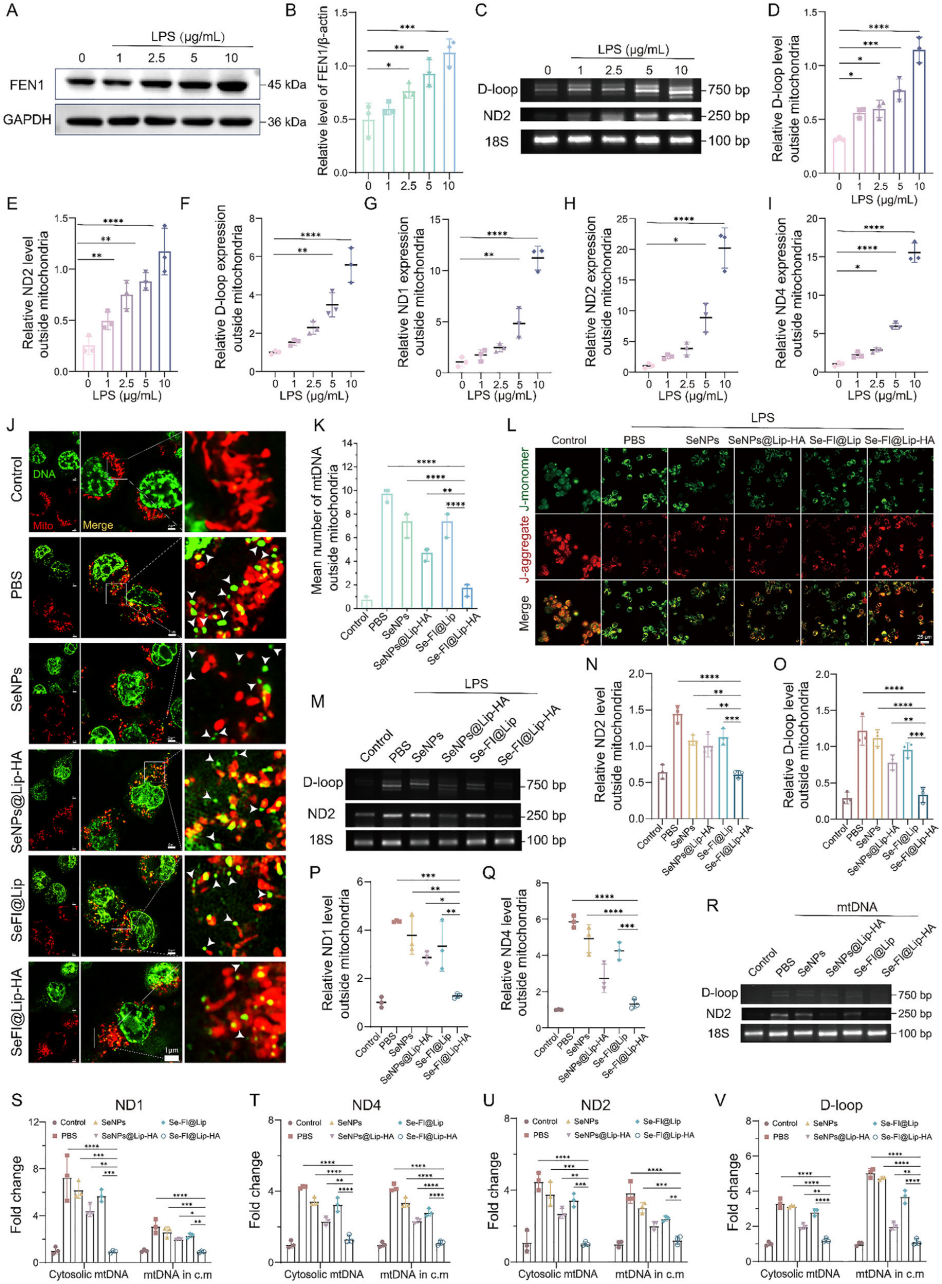
FI inhibits the release of mtDNA fragments and preserves mitochondrial homeostasis. (A) WB assay of FEN1 expression in RAW264.7 treated with LPS of different concentrations (1, 2.5, 5, 10 µg/mL), representative images from *n*  =  3 independent experiments. (B) Quantitative analysis of FEN1 WB results. (C) Detection of cytosolic mtDNA by PCR with D‐loop and ND2 primers after incubation with different concentrations of LPS. (D, E) Quantitative results of D‐loop and ND2 bands in PCR assays (*n* = 3). (F–I) Detection of mtDNA content in RAW264.7 cytoplasm via qPCR with D‐loop, ND1, ND2, and ND4 primers after treatment with different concentrations of LPS (*n* = 3). (J) Colocalization analysis of mtDNA and mitochondria in RAW264.7 via Polar‐SIM after inflammatory induction and treatment with different formulations. mtDNA was labeled with Picogreen (green), and mitochondria were labeled with MitoTracker Red (red). Scale bar: 2 µm. (K) Quantification of mtDNA outside mitochondria corresponding to (J). (L) CLSM images of MMP detection. Scale bar: 25 µm. (M–O) Detection and quantification of mtDNA in inflamed RAW264.7 cells treated with different formulations by PCR with D‐loop and ND2 primers (*n* = 3). (P, Q) Determination of cytosolic mtDNA content in inflamed RAW264.7 cells treated with different formulations via qPCR with ND1 and ND4 primers (*n* = 3). (R) Determination of mtDNA content in cell culture medium via PCR. (S–V) Determination of mtDNA content in cytoplasm and culture medium of inflamed RAW264.7 cells treated with different formulations via qPCR (*n* = 3). Data are presented as mean ± SD and analyzed by one‐way ANOVA with Tukey's multiple comparisons test. *p < 0.05, **p < 0.01, and ***p < 0.001, ****p < 0.0001.

Previous studies have shown that in LPS‐primed macrophages, mitochondrial permeability transition pore (mPTP) opening and voltage‐dependent anion channel (VDAC) oligomerization provide a physical channel for the release of mtDNA. Notably, Ox‐mtDNA fragments may preferentially pass through these channels, and this fragmentation is governed by mitochondrial FEN1‐mediated cleavage [18, 22]. We systematically investigated the expression of FEN1 and cytosolic mtDNA levels in RAW264.7 under different concentrations of LPS stimulation. Notably, FEN1 protein expression was enhanced by LPS priming, which increased with LPS concentration (Figure 5A,B). This would promote cleavage of mitochondrial DNA. Under oxidative stress, Ox‐mtDNA fragments are released into the cytosol [52]. Accordingly, we extracted the cytosolic DNA and detected by mitochondrial genes D‐loop and ND2 primers using PCR, and gel electrophoresis showed notable mtDNA release into the cytosol under stress with increasing LPS concentrations (Figure 5C–E). Furthermore, the associated mtDNA in the cytoplasm including D‐loop, ND1, ND2, and ND4, was measured by qPCR (Figure 5F–I). These results further demonstrate increased accumulation of cytosolic mtDNA fragments in LPS‐primed cells.

We next investigated the inhibitory capability of Se‐FI@Lip‐HA on cytosolic mtDNA in RAW264.7 cells pre‐stimulated by LPS. Dual immunostaining of Mito and DNA revealed that the amount of mtDNA escaping into the cytosol was significantly restrained by Se‐FI@Lip‐HA compared with other treatments (Figure 5J,K and Figure S18). Subsequently, we analyzed the distribution and the level of mtDNA fragments in the cytoplasm using agarose gel electrophoresis. The results showed that the Se‐FI@Lip‐HA treatment significantly reduced the level of cytoplasmic mtDNA fragments compared to the LPS and SeNPs@Lip‐HA groups (Figure S19). To quantitatively analyze the levels of mtDNA in the cytoplasm after different treatments, cytosolic DNA was separated and transferred to PCR analysis using primers specific to the ND2 and D‐loop genes. The level of mtDNA was undetectable by PCR in unstimulated cells and significantly increased after LPS stimulation. After Se‐FI@Lip‐HA treatment, the relative cytosolic mtDNA level was notably reduced (Figure 5M–O). We further assessed cytosolic genes, including ND1, ND2, ND4, and D‐loop expression using qPCR (Figure 5P,Q and Figure S20). This observation was consistent with colocalization analysis and PCR results. These findings suggested that Se‐FI@Lip‐HA potently inhibits the exposure of mtDNA in the cytoplasm. The effect of Se‐FI@Lip‐HA may be attributed to the inhibition of mitochondrial FEN1 by FI, which in turn prevents mtDNA cleavage and thereby reduces cytosolic mtDNA levels. Notably, the mitochondrial membrane is the physical barrier of mtDNA, and the destruction of its integrity will cause the efflux of mtDNA [53]. Mitochondrial membrane potential (MMP) is a sensitive indicator of membrane integrity; we further used the JC‐1 fluorescent dye to assess MMP [54]. In the control group, JC‐1 aggregates form and emit red fluorescence. In contrast, after LPS stimulation, the green fluorescence of monomers in the PBS group was higher. The value of red/green fluorescence intensity after Se‐FI@Lip‐HA treatment was obviously higher than other formulations (Figure 5L and Figure S21). Together, these results suggested that Se‐FI@Lip‐HA not only inhibits the release of mitochondrial DNA but also promotes mitochondrial homeostasis, which indicated a favorable guarantee to prevent mtDNA escaping into the cytoplasm.

**FIGURE 6:**
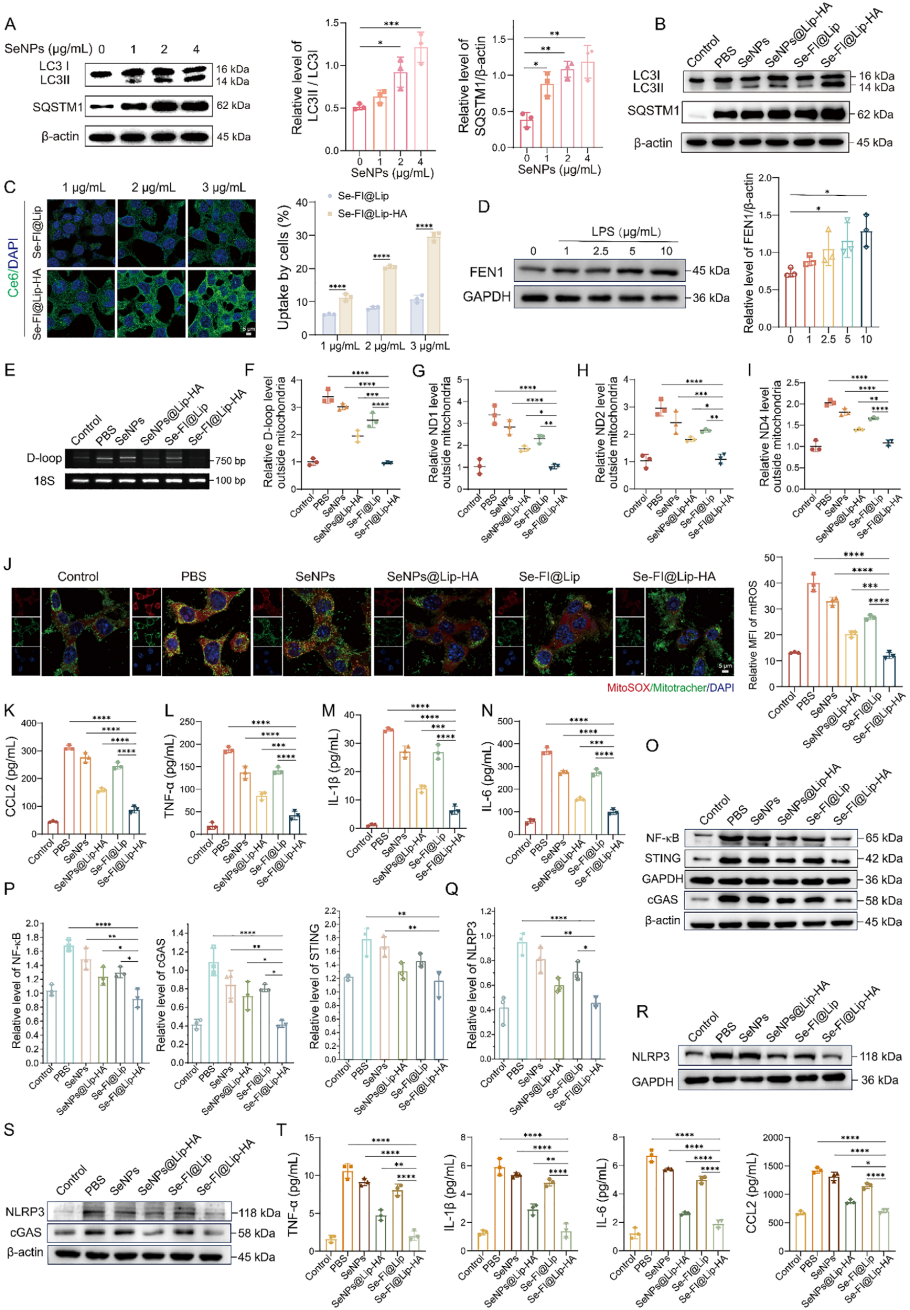
Anti‐inflammatory effects in macrophages and epithelial cells. (A) Representative WB results and quantitative analysis of LC3I, LC3II, and SQSTM1 in RAW264.7 treated with SeNPs at different concentrations. (B) Representative WB results of LC3I, LC3II, and SQSTM1 in RAW264.7 cells treated with different formulations. (C) CLSM images and quantitative analysis of the uptake of Ce6‐labeled Se‐FI@Lip and Se‐FI@Lip‐HA at different concentrations by MLE‐12 cells (*n* = 3). (D) WB assay and quantification of FEN1 expression in MLE‐12 cells treated with LPS of different concentrations (*n* = 3). (E) Detection and quantification of cytoplasmic mtDNA in inflamed MLE‐12 cells treated with different formulations via PCR with D‐loop primers. (F–I) Determination of extramitochondrial mtDNA content in inflamed MLE‐12 cells treated with different formulations via qPCR (*n* = 3). (J) CLSM images of mtROS and quantitative analysis of fluorescence intensity in MLE‐12 cells after stimulation by LPS. mtROS was labeled with MitoSOX (red), and mitochondria were labeled with MitoTracker Green (green). Scale bar: 5 µm. (K–N) Concentration analysis of cytokines CCL2, TNF‐α, IL‐1β, and IL‐6 in the medium of inflamed RAW264.7 cells treated with different formulations. (O) Representative WB results of NF‐κB, STING, and cGAS in RAW264.7 cells treated with various experimental groups. (P) Quantitative analysis of NF‐κB, STING, and cGAS corresponding to WB results (*n* = 3). (Q, R) Representative WB results and quantitative analysis of NLRP3 in RAW264.7 treated with different experimental groups (*n* = 3). (S) Representative WB results of NLRP3 and cGAS in MLE‐12 cells treated with different experimental groups. (T) Levels of TNF‐α, IL‐1β, IL‐6 and CCL2 in the culture medium of MLE‐12 cells treated with different formulations after LPS stimulation (*n* = 3). Data are presented as mean ± SD and analyzed by one‐way ANOVA (A, B, D, F‐I, J, K‐N, P, Q, T) and two‐way ANOVA (C) with Tukey's multiple comparisons test. *p < 0.05, **p < 0.01, and ***p < 0.001, ****p < 0.0001.

Next, we evaluated the capacity of Se‐FI@Lip‐HA to eliminate extracellular mtDNA. Cytosolic DNA was isolated from LPS‐primed macrophages and used to stimulate RAW264.7 cells. Different formulations were then added for treatment. We analyzed the mtDNA levels in the cell culture medium by PCR, and the gel electrophoresis results showed that SeNPs@Lip‐HA and Se‐FI@Lip‐HA significantly reduced the amount of extracellular mtDNA compared to other groups (Figure 5R). This may be attributed to the ability of SeNPs to promote endocytosis in macrophages [55]. As damage‐associated molecular patterns (DAMPs), mtDNA is endocytosed by macrophages and activates the TLR9 pathway, thereby promoting inflammatory responses [56]. Therefore, we further investigated the cytoplasmic and extracellular mtDNA levels in mtDNA‐primed RAW264.7 cells by qPCR (Figure 5S–V). Interestingly, the level of cytosolic mtDNA increased under mtDNA stimulation and decreased after Se‐FI@Lip‐HA treatment. This might be because SeNPs promote autophagy and enhance cytosolic mtDNA clearance [28], while FI inhibits mitochondrial mtDNA cleavage and further reduces mtDNA release. Subsequently, we investigated whether the introduction of SeNPs could improve autophagy in RAW264.7 cells. The expression of autophagy‐related protein sequestosome 1 (SQSTM1) and the LC3II/LC3I ratio was increased with SeNPs concentration (Figure 6A). In addition, Se‐FI@Lip‐HA promoted SQSTM1 expression and the LC3II/LC3I ratio in RAW264.7 cells (Figure 6B). Lysosomal inhibitor chloroquine (CQ) further demonstrated that Se‐FI@Lip‐HA treatment significantly increased autophagy‐related protein levels. These results indicate that the nanoplatform promotes autophagic flux rather than blocking autophagosome degradation (Figure S22). Generally, our findings highlight an effective approach for clearing extracellular mtDNA and limiting its pathogenic role in inflammation.

### 2.5 Anti‐Inflammatory Effects in Macrophages and Epithelial Cells

The above‐mentioned Ox‐mtDNA fragments escaping from stressed mitochondria would result in activation of three different pattern recognition receptors (PRRs), leading to inflammatory eruption [24, 56]. The anti‐inflammatory capability of Se‐FI@Lip‐HA in vitro was evaluated in RAW264.7 and MLE‐12. Firstly, we evaluated the uptake capacity of MLE‐12 cells for Se‐FI@Lip‐HA. Fluorescence image results showed that LPS‐primed MLE‐12 treated with Se‐FI@Lip‐HA exhibited a higher fluorescence intensity in a concentration‐dependent manner (Figure 6C). This could result from the increased CD44 expression on MLE‐12 after LPS stimulation.

We further investigated the FEN1 expression of MLE‐12 after LPS priming at different concentrations. WB assay demonstrated that FEN1 expression is enhanced with increased LPS concentration (Figure 6D). Subsequently, cytoplasmic mtDNA in MLE‐12 treated with different formulations was extracted and detected by PCR. The level of D‐loop was significantly increased in LPS‐primed MLE‐12 and decreased after Se‐FI@Lip‐HA treatment (Figure 6E). In addition, cytosolic mtDNA including D‐loop, ND1, ND2, and ND4, was quantified by qPCR. The results further demonstrated that Se‐FI@Lip‐HA significantly restrains the release of mtDNA into the cytoplasm (Figure 6F–I). These findings suggested that under LPS stimulation, mtDNA exposure in the cytoplasm of MLE‐12 is increased, which could be effectively blocked by Se‐FI@Lip‐HA. Next, we investigated the antioxidant properties of Se‐FI@Lip‐HA in MLE‐12. As shown in Figure 6J, the fluorescence of MitoSOX was elevated in LPS‐stimulated MLE‐12, and treatment with Se‐FI@Lip‐HA reduced the ROS level. These results indicated the potential of Se‐FI@Lip‐HA to inhibit cytosolic Ox‐mtDNA production in epithelial cells.

Ox‐mtDNA fragments escaping to the cytoplasm ignite the cGAS‐STING pathway and the NLRP3 inflammasome, which subsequently activate two key inflammatory cascades, NF‐κB and IRF3. This leads to the production of pro‐inflammatory cytokines, including type I interferons, TNF‐α, IL‐1β, and chemokines [57, 58]. Thus, we researched the intracellular anti‐inflammatory properties of Se‐FI@Lip‐HA. The cytokine content in the medium of RAW264.7 after different treatments was estimated by ELISA (Figure 6K–N). The results revealed that the levels of the inflammatory factors TNF‐α, IL‐1β, and IL‐6 in the Se‐FI@Lip‐HA group were remarkably decreased. Meanwhile, the level of chemokine CCL2 was also significantly reduced in the Se‐FI@Lip‐HA group. Additionally, we evaluated the anti‐inflammatory effects of Se‐FI@Lip‐HA at various concentrations. As shown in Figure S23, the content of related cytokines in the medium of inflamed RAW264.7 was markedly reduced after co‐incubation with Se‐FI@Lip‐HA, and its anti‐inflammatory effect increased with increasing Se‐FI@Lip‐HA concentration. To further investigate the anti‐inflammatory mechanism, WB analysis was performed to examine mtDNA‐activated downstream proteins, including cGAS and NLRP3, in RAW264.7 cells. Downstream proteins were obviously increased after LPS stimulation, and the introduction of Se‐FI@Lip‐HA significantly inhibited the expression of inflammatory pathway proteins (Figure 6O–R). To functionally validate pathway inhibition, we measured cGAMP levels and caspase‐1 activity in treated macrophages. As shown in Figure S25, Se‐FI@Lip‐HA significantly decreased both cGAMP levels and caspase‐1 activity, consistent with the change of downstream effector cytokines. Taken together, these results demonstrate that Se‐FI@Lip‐HA effectively inhibits both the cGAS‐STING and NLRP3 pathways.

Early epithelial damage compromises alveolar barrier integrity, promotes the release of mitochondrial danger signals, and amplifies inflammatory cascades in ARDS. Next, we determined whether Se‐FI@Lip‐HA exerts anti‐inflammatory effects in MLE‐12. Of note, the results indicated that downstream proteins, chemokines, and inflammatory factors were remarkably elevated in LPS‐stimulated MLE‐12. After Se‐FI@Lip‐HA treatment, the expression of intracellular cGAS and NLRP3 was significantly down‐regulated and the levels of TNF‐α, IL‐1β, IL‐6, and CCL2 in the medium were also remarkably decreased (Figure 6S,T and Figure S24). These results demonstrated the effective anti‐inflammatory effect of Se‐FI@Lip‐HA in macrophages and epithelial cells.

Extracellular cf‐mtDNA endocytosed by macrophages activates TLR9, triggering inflammatory responses [52]. To further investigate the influence of cf‐mtDNA on macrophages and the anti‐inflammatory effects of Se‐FI@Lip‐HA, cytosolic mtDNA was isolated and used to stimulate RAW264.7 cells, followed by different treatments. As shown in Figure S26A,B, the expression of TLR9 was increased under mtDNA stimulation, which was significantly downregulated after Se‐FI@Lip‐HA treatment. Consistently, stimulation of mtDNA increased associated inflammatory factors, while Se‐FI@Lip‐HA successfully suppressed the expression of TNF‐α and IL‐6 (Figure S26C). Inspired by the above observations, we further investigated the anti‐inflammatory effects of different nanoparticles on epithelial cells under mtDNA stimulation. Interestingly, similar results were observed in mtDNA‐primed MLE‐12 (Figure S27). These findings demonstrated that Se‐FI@Lip‐HA also has a potent inhibitory effect on the inflammatory response caused by extracellular mtDNA stimulation. Additionally, studies have shown that downregulation of inflammatory factors promotes the polarization of activated macrophages (M1) to M2 with anti‐inflammatory functions [59]. Consequently, we further assessed the polarization degree of RAW264.7 cells after different treatments. As shown in Figure S28, the highest red fluorescence of M2 was observed in Se‐FI@Lip‐HA groups, whereas green fluorescence of M1 was notably reduced. This finding further suggested that Se‐FI@Lip‐HA has great potential in anti‐inflammatory therapy.

### 2.6 Se‐FI@Lip‐HA Inhibits Inflammation in a Mouse Model of ARDS

**FIGURE 7:**
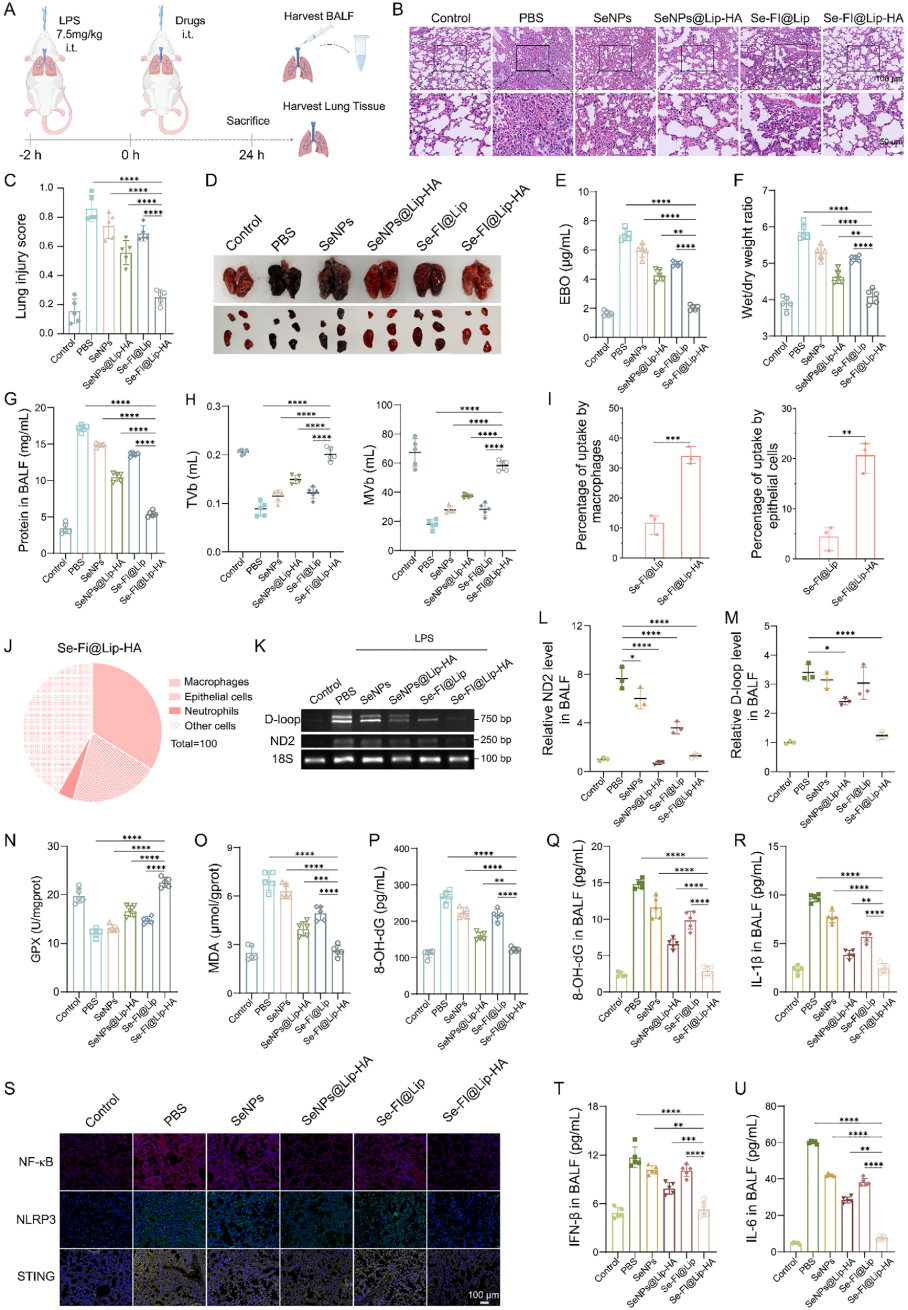
Anti‐inflammatory effects of Se‐FI@Lip‐HA in a mouse model of ARDS. (A) Schematic schedule of modeling and drug administration in ARDS mice. (B) H&E staining of histological images in lung sections from mice in different treatment groups. Scale bars in the panels are 100 and 50 µm, respectively. (C) Total lung injury score determined by five pathophysiological indices (alveolar neutrophil, interstitial neutrophil, hyaline membrane, proteinaceous debris, alveolar septal thickening scores) based on histological images (*n* = 5). D) Lung images at 2 h post intravenous injection of Evans blue dye after different group treatments. (E) Quantification of Evans blue dye content in the lung (*n* = 5). (F) Lung W/D weight ratio (*n* = 5). (G) The protein concentration in BALF (*n* = 5). (H) Measurement of lung function indicators, including tidal volume (TVb) and minute ventilation (MVb) (n = 5). (I) Flow cytometry analysis of the uptake of DiI‐labeled Se‐FI@Lip and Se‐FI@Lip‐HA by macrophages and epithelial cells in lung single‐cell suspensions (*n* = 3). (J) Uptake distribution of DiI‐labeled Se‐FI@Lip‐HA in various lung cells. (K) Detection of mtDNA content in BALF by PCR using D‐loop and ND2 primers. (L, M) Quantification of mtDNA content in BALF via qPCR using ND2 and D‐loop primers (*n* = 3). (N–P) Quantification of GPX, MDA, and 8‐OH‐dG levels in lung tissues (*n* = 5). (Q, R) Levels of 8‐OH‐dG and IL‐1β in BALF (*n* = 5). (S) Representative immunofluorescence images of the lung tissues from ARDS model mice stained with NF‐κB (red), NLRP3 (green), and STING (yellow) in different treatment groups. Scale bar: 100 µm. (T, U) Levels of IFN‐β and IL‐6 in BALF (*n* = 5). Data are presented as mean ± SD and analyzed via one‐way ANOVA with Tukey's multiple comparisons test. *p < 0.05, **p < 0.01, and ***p < 0.001, ****p < 0.0001.

Given the excellent anti‐inflammatory performance achieved by Se‐FI@Lip‐HA in RAW264.7 and MLE‐12 cells, we investigated the therapeutic potential of Se‐FI@Lip‐HA for inflammatory diseases in the ARDS mouse model. As illustrated in Figure 7A, at 2 h post LPS endotracheal stimulation, mice received different treatments by endotracheal administration, and the therapeutic efficacy was analyzed after 24 h. Prior to efficacy studies, we assessed the pulmonary targeting and retention in a mouse model of ARDS. DiI‐labeled SeNPs@Lip‐HA, Se‐FI@Lip, and Se‐FI@Lip‐HA were administered intratracheally in vivo. The fluorescence imaging of major organs showed that SeNPs@Lip‐HA and Se‐FI@Lip‐HA exhibited higher fluorescence intensity in the lung regions compared to the Se‐FI@Lip group (Figure S29A,B). The results indicated that the modification of HA might enhance the uptake of pulmonary cells, which enhanced the retention of the nanoparticles in the lungs. To examine the uptake proportion of Se‐FI@Lip‐HA in different cell types, we extracted the cell suspensions from lung tissue and analyzed the colocalization by flow cytometry. Strikingly, Se‐FI@Lip‐HA was primarily internalized by macrophages and epithelial cells, and the uptake percentage was higher in both cell types compared with Se‐FI@Lip (Figure 7I,J). Following this, the therapeutic efficacy was evaluated after different treatments in the LPS‐induced ARDS model. H&E staining and lung injury score demonstrated that Se‐FI@Lip‐HA markedly reduced lung damage (Figure 7B,C and Figure S30). Pulmonary vascular leakage also improved significantly after Se‐FI@Lip‐HA treatment (Figure 7D,E). Pulmonary vascular leakage induces edema fluid and protein leaks into the alveoli, which results in pulmonary edema [60]. Wet‐to‐dry weight (W/D) ratio was measured to assess the degree of pulmonary edema. Compared to other groups, pulmonary edema in the Se‐FI@Lip‐HA group was notably alleviated (Figure 7F). Meanwhile, we collected BALF to investigate protein concentration and cell count. Consistently, the group treated with Se‐FI@Lip‐HA displayed negligible elevation of cell and protein content compared to the normal group (Figure 7G and Figure S31A). These results indicated that Se‐FI@Lip‐HA effectively mitigates LPS‐induced lung tissue damage. Subsequently, pulmonary function was detected after different treatments. As shown in Figure 7H and Figure S32, respiration parameters regressed to normal values after Se‐FI@Lip‐HA treatment, which further demonstrated the excellent therapeutic efficacy of Se‐FI@Lip‐HA in ARDS.

To further investigate the therapeutic mechanism of Se‐FI@Lip‐HA, we analyzed the level of leaked mtDNA in different treatment groups. According to PCR and qPCR analysis, the mtDNA level in BALF was dramatically increased in ARDS mice, while it decreased significantly under Se‐FI@Lip‐HA treatment (Figure 7K–M and Figure S31B). Autophagy‐related protein expression aligns with the outcomes of mtDNA level in BALF (Figure S33A). Meanwhile, ROS level was examined by immunofluorescence analysis. As depicted in Figure S33A, the red fluorescence intensity associated with ROS is considerably weaker in Se‐FI@Lip‐HA than in other groups. Additionally, following Se‐FI@Lip‐HA treatment, the level of the antioxidant enzymes GPX and SOD was dramatically elevated, while MPO responsible for producing ROS was significantly reduced (Figure 7N and Figure S33A,B). Consequently, lipid peroxidation damage of lung tissue in the Se‐FI@Lip‐HA group was improved (Figure 7O). Based on the above observations, we investigated the amount of Ox‐mtDNA following various treatments. The level of 8‐OH‐dG was significantly reduced in both BALF and lung homogenate after Se‐FI@Lip‐HA treatment (Figure 7P,Q). These results demonstrated that Se‐FI@Lip‐HA effectively inhibits the generation of Ox‐mtDNA in vivo. Subsequently, we evaluated downstream protein expression and the production of inflammatory factors. Consistent with the Ox‐mtDNA findings, Se‐FI@Lip‐HA greatly inhibited the activation of inflammatory pathway proteins and the production of related inflammatory factors (Figure 7R–U and Figure S34). Notably, CXCL1, as a neutrophil chemokine, was decreased. Thus, we further analyzed the levels of neutrophils in BALF of different groups. Flow cytometry analysis and Giemsa‐Wright staining showed that neutrophil recruitment was significantly reduced after Se‐FI@Lip‐HA treatment (Figure S35). Together, these results suggested that Se‐FI@Lip‐HA effectively inhibits the leakage of Ox‐mtDNA and blocks subsequent inflammatory outbreaks.

The downregulation of inflammatory reaction promotes the polarization of macrophages toward the M2 phenotype, which boosts resolution of inflammation. Flow cytometry analysis and immunofluorescence images indicated that the Se‐FI@Lip‐HA group showed a higher level of M2. The anti‐inflammatory cytokine IL‐10 secreted by M2 macrophages was also significantly increased following Se‐FI@Lip‐HA treatment (Figure S36). These findings further confirmed the effective anti‐inflammatory properties of Se‐FI@Lip‐HA. Next, the safety of different nanoparticles was assessed in vivo. The main organs H&E staining showed that there were no discernible pathological anomalies or damage indicators in all groups (Figure S37A). Furthermore, whole blood and serum were collected for blood routine and blood biochemistry analysis. As shown in Figure S37B,C, blood routine index values were within the normal range in all groups, and there was no notable disparity in liver and kidney function between the different groups. Taken together, the results demonstrated that Se‐FI@Lip‐HA did not induce systemic toxicity in mice, which provides a strong guarantee for the biological application of Se‐FI@Lip‐HA in inflammatory diseases.

### 2.7 Expanded Application of Se‐FI@Lip‐HA in a Sepsis‐Induced ARDS Model

**FIGURE 8:**
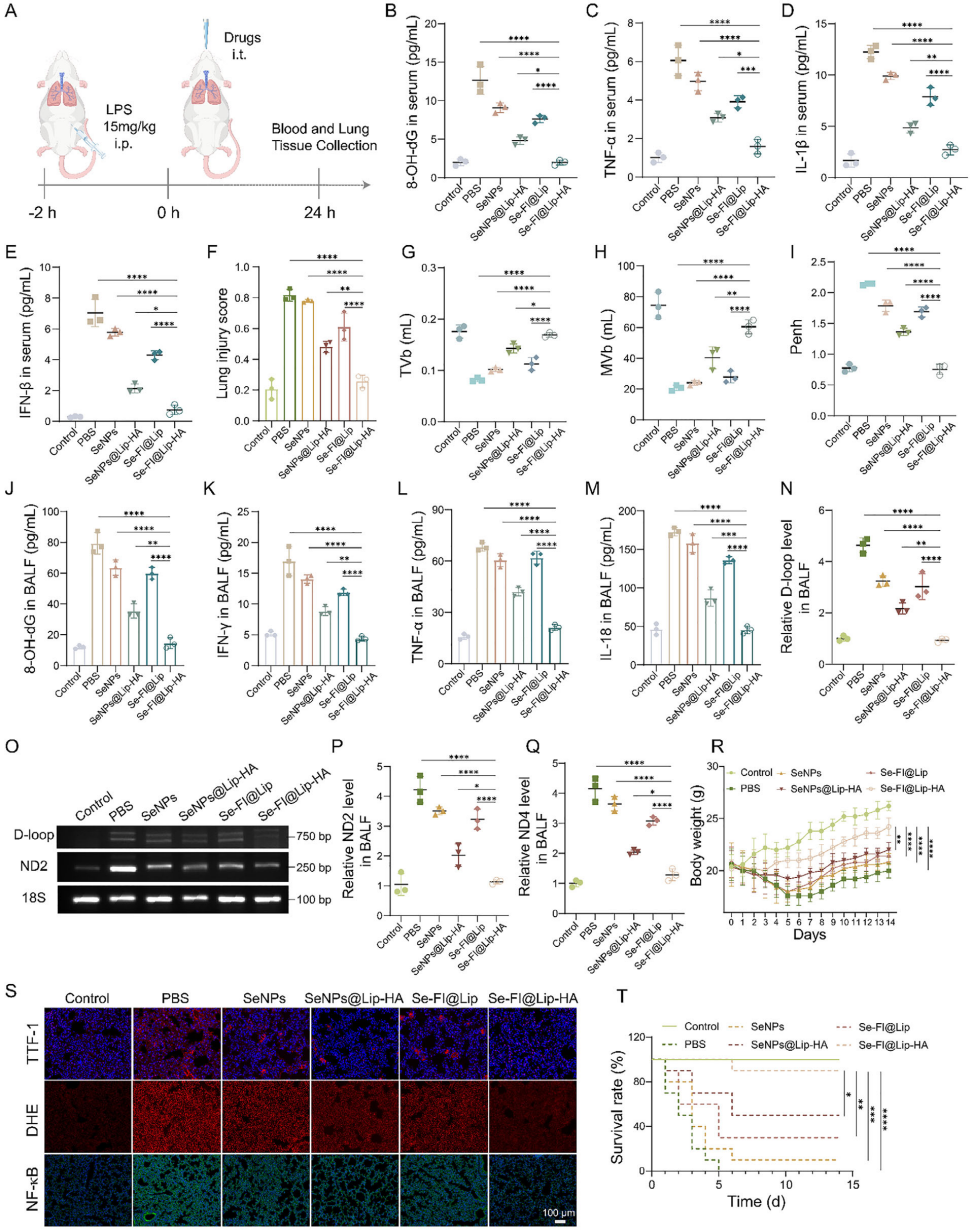
Anti‐inflammatory effects of Se‐FI@Lip‐HA in sepsis‐induced ARDS. (A) Schematic schedule of modeling and drug administration in sepsis‐induced ARDS mice. (B–E) Levels of 8‐OH‐dG, TNF‐α, IL‐1β, and IFN‐β in serum. (*n* = 3). (F) Lung injury score of sepsis‐induced ARDS mice in different treatment groups, based on H&E‐stained lung sections (*n* = 3). (G–I) Measurement of lung function indicators, including TVb, MVb, and Penh (*n* = 3). (J–M) Levels of 8‐OH‐dG, IFN‐γ, TNF‐α and IL‐18 in BALF (*n* = 3). (N) Quantification of mtDNA content in BALF via qPCR using D‐loop primer (*n* = 3). (O) mtDNA content in BALF via PCR using D‐loop and ND2 primers. (P, Q) Quantification of mtDNA content in BALF via qPCR using ND2 and ND4 primers (*n* = 3). (R) Body weight changes in sepsis‐induced ARDS mice over 14 days after treatment (*n* = 5). (S) Representative immunofluorescence images of the lung tissues from sepsis‐induced ARDS model mice stained with TTF‐1 (red), DHE (red) and NF‐κB (green) in different treatment groups. Scale bar: 100 µm. (T) Survival curves of sepsis‐induced ARDS mice within 14 days after administration in different treatment groups. Data are presented as mean ± SD and analyzed by one‐way ANOVA with Tukey's multiple comparisons test. *p < 0.05, **p < 0.01, and ***p < 0.001, ****p < 0.0001.

Inspired by the therapeutic efficacy of Se‐FI@Lip‐HA in the ARDS mice, we investigated its potential to inhibit the generation of Ox‐mtDNA for the treatment of a broader range of inflammatory diseases. Sepsis‐induced ARDS reflects a systemic inflammatory milieu and provides a more clinically relevant platform to evaluate Ox‐mtDNA–targeted interventions. We established an LPS‐induced endotoxemia model by intraperitoneal injection of LPS to mimic sepsis‐associated acute lung injury. The lung is one of the organs most commonly affected during sepsis, and sepsis‐associated lung injury can clinically manifest as ARDS [61]. To this end, we evaluated the anti‐inflammatory efficacy of Se‐FI@Lip‐HA in a sepsis‐induced ARDS mouse model (Figure 8A). In sepsis, serum levels of inflammatory cytokines such as TNF‐α, IL‐1β, and IFN‐β increased significantly, which indicated the occurrence of systemic inflammatory response. Se‐FI@Lip‐HA markedly reduced the level of these inflammatory factors and significantly reduced circulating Ox‐mtDNA in serum (Figure 8B–E). Meanwhile, H&E staining of lung tissues demonstrated that sepsis induced severe pulmonary inflammatory cell infiltration, protein debris deposition, and alveolar damage (Figure S38). Fortunately, after treatment with Se‐FI@Lip‐HA, lung injury was significantly alleviated, and the injury score was markedly reduced (Figure 8F). We further evaluated the respiratory function of mice in each group. The results showed that, in comparison with non‐targeted SeNPs, Se‐FI@Lip, and FI‐free SeNPs@Lip‐HA, Se‐FI@Lip‐HA effectively restored the pulmonary function of mice, including the tidal volume (TV), minute volume (MV), and pause enhanced (Penh) (Figure 8G–I). Next, we collected BALF to determine the levels of inflammatory factors and Ox‐mtDNA. ELISA results showed that, compared with the PBS group, Se‐FI@Lip‐HA significantly reduced the levels of pro‐inflammatory cytokines and the level of 8‐OH‐dG (Figure 8J–M). Consistently, Se‐FI@Lip‐HA also significantly alleviated pulmonary neutrophil infiltration and ameliorated pulmonary inflammation (Figure S39A). Furthermore, we investigated the content of mtDNA in BALF; the results showed that the level of circulating mtDNA in BALF was significantly elevated in septic mice, whereas this level was markedly reduced after treatment with Se‐FI@Lip‐HA, demonstrating its potential to inhibit the leakage of mtDNA fragments in sepsis‐induced ARDS mice (Figure 8N–Q, Figure S39B). Levels of ALT, AST, and BUN in serum were measured to evaluate liver and kidney injury. Meanwhile, CBC was performed to assess the core functional status of the hematological system. The results showed that mice in the PBS group exhibited severe liver and kidney injury, accompanied by a significant increase in white blood cell count and a marked decrease in platelet count. In contrast, treatment with Se‐FI@Lip‐HA alleviated liver and kidney injury and suppressed the elevation of WBC (Figure S40). Survival rate assessment showed that all mice in the PBS group died within 5 days post‐administration, whereas treatment with Se‐FI@Lip‐HA increased the survival rate to 90% (Figure 8T). Meanwhile, the body weight of surviving mice gradually recovered over the 14‐day treatment period (Figure 8R).

Based on the preliminary findings mentioned above in vitro, Se‐FI@Lip‐HA could effectively scavenge reactive oxygen species (ROS). We further investigated its ROS‐scavenging capacity in sepsis‐induced ARDS mice via DHE staining of lung tissues, and the results showed that Se‐FI@Lip‐HA exerted a potent ROS‐scavenging effect, whereas non‐targeted SeNPs, Se‐FI@Lip, and FI‐free SeNPs@Lip exhibited limited ROS‐scavenging activity (Figure 8S). Consistently, Se‐FI@Lip‐HA also significantly upregulated the pulmonary expression of GPX4 to enhance antioxidant capacity, and markedly inhibited MPO‐mediated oxidative stress (Figure S41). Furthermore, it significantly elevated the expression of autophagy‐related proteins (Figure S42), which promoted the removal of released mtDNA fragments and prevented the activation of the NLRP3 and NF‐κB pathways (Figure 8S, Figure S41). Meanwhile, Se‐FI@Lip‐HA effectively reprogrammed pulmonary M1 macrophages into an anti‐inflammatory phenotype (Figure S42B), and preserved pulmonary epithelial cell marker expression (Figure 8S). Collectively, these results indicate that Se‐FI@Lip‐HA mitigates sepsis‐associated inflammatory progression by reducing Ox‐mtDNA exposure and preventing excessive inflammatory activation.

## 3 Conclusion

Inflammatory diseases are one of the main culprits of human health, and persistent or excessive inflammatory responses lead to tissue damage, organ failure, and even death [32, 33]. However, the majority of inflammatory diseases currently lack effective treatments. In the progression of inflammation, Ox‐mtDNA fragments play a particularly critical role. Ox‐mtDNA not only enhances the ability of mtDNA to activate TLR9 inflammatory pathways, but is also a powerful activator of the cGAS‐STING pathway and more effectively activates NLRP3 inflammasomes [24, 25, 62]. Therefore, developing strategies to selectively suppress Ox‐mtDNA formation is critical for achieving safe and effective therapies against inflammatory diseases. Inspired by the application of FEN1 inhibitors in tumors, which mainly interfere with nuclear FEN1 function [27, 63], we reported a mitochondrial‐targeted FEN1 inhibitor FI, which targets mitochondria and inhibits the cleavage of mtDNA by FEN1, thereby blocking the formation of mtDNA fragments. Here, we have shown that FI docks with FEN1 and has lower cytotoxicity. Meanwhile, SeNPs scavenge ROS to reduce the Ox‐mtDNA level and promote macrophage phagocytosis and autophagy to further clear extracellular mtDNA. FI and SeNPs were co‐loaded into hyaluronic acid (HA)‐functionalized lipid nanoparticles for targeted delivery to inflammatory cells.

We initially examined the difference in endonuclease expression, Ox‐mtDNA accumulation, the distribution of mtDNA fragments, and cytosolic DNA‐mediated inflammatory signaling in lung tissues from control and ARDS groups using RNA sequencing, immunofluorescence staining, and gel electrophoresis. Following LPS challenge, the expression of endonucleases, including FEN1, as well as ROS‐producing genes, was markedly upregulated, which was accompanied by increased mtDNA oxidation, enhanced generation of mtDNA fragments, and their aberrant accumulation in the cytosol. These changes collectively promoted robust activation of inflammatory pathways ignited by Ox‐mtDNA, providing a mechanistic basis for the exacerbated inflammatory response observed in ARDS.

The therapeutic mechanism of Se‐FI@Lip‐HA was investigated in macrophages and epithelial cells in vitro. We conducted a series of assays including nucleic acid gel electrophoresis, PCR, qPCR, and ELISA to quantify the amount of mtDNA in the cytoplasm after different treatments. In addition, anti‐inflammatory efficacy was evaluated by examining downstream signaling proteins and proinflammatory cytokine levels. Together, these results indicate that Se‐FI@Lip‐HA obviously suppressed Ox‐mtDNA formation and leakage, thereby mitigating downstream inflammatory activation. Importantly, in both a typical ARDS model and a sepsis‐induced ARDS model, Se‐FI@Lip‐HA effectively alleviated inflammatory outbreaks and lung damage. This strategy may serve as a universal approach to treating inflammatory diseases where Ox‐mtDNA fragments are continually released and cannot be eliminated.

In summary, our work provides a proof of concept for blocking the ignition of Ox‐mtDNA fragments to inflammation by Se‐FI@Lip‐HA. Beyond offering a promising strategy for the treatment of ARDS, this strategy may serve as a broadly applicable approach and warrants further investigation in other inflammatory disease models associated with pathological Ox‐mtDNA release.

## 4 Materials and Methods

### 4.1 Materials and Reagents

Phospholipid and DSPE‐mPEG_2K_ were purchased from AVT Pharmaceutical Tech Co., Ltd. (Shanghai, China). Cholesterol was obtained from Sinopharm Chemical Reagent Co., Ltd. (Beijing, China). L‐Ascorbic acid was purchased from J Scientific Co., Ltd. (Beijing, China), and Chitosan from Jinan Haidebei Marine Bioengineering Co., Ltd. (Jinan, China). Sodium selenite (Na_2_SeO_3_), Hyaluronic acid (HA), and N‐hydroxysuccinimide (NHS) were purchased from Shanghai Aladdin Biochemical Technology Co., Ltd. (Shanghai, China). Ethyl dimethylaminopropyl Carbodiimide (EDC) was purchased from Shanghai Macklin Biochemical Co., Ltd. (Shanghai, China). Chlorin e6 (Ce6) was obtained from Tokyo Kasei Co., Ltd. (Tokyo, Japan), and DiI was from Shanghai Bioscience Co. Ltd. (Shanghai, China). BSA and Cell Counting Kit‐8 (CCK‐8) were purchased from Biosharp Company (Hefei, China). MTT was purchased from Guangzhou Saiguo Biotech Co., Ltd. (Guangzhou, China). Total Antioxidant Capacity Assay Kit with ABTS method (S0119), Hydrogen Peroxide Assay Kit (S0038), Cell Mitochondria Isolation Kit (S3601), Enhanced mitochondrial membrane potential assay kit with JC‐1 (C20035), DNA loading buffer (D0072) and Alexa Fluor 647‐labeled Goat Anti‐Mouse IgG (H + L) (A0473) were obtained from Beyotime Biotechnology Co., Ltd. (Shanghai, China), DPPH Free Radical Scavenging Capacity Assay Kit (A153‐1‐1) and Inhibition and produce superoxide anion assay kit (A052‐1‐1) were purchased from Nanjing Jiancheng Technology Co., Ltd. (Nanjing, China). Fetal bovine serum (FBS) was acquired from Gibco Life Technologies (New York, USA). Lipopolysaccharide (LPS) and Tetramethylbenzidine (TMB) were purchased from Sigma–Aldrich Corporation (St. Louis, MO, USA). Dulbecco's modified Eagle's medium (DMEM), Dulbecco's Modified Eagle Medium/Nutrient Mixture F12 Ham (DMEM/F12), RPMI‐1640 medium, phosphate‐buffered saline (PBS), 4,6‐diamidino‐2‐phenylindole dihydrochloride (DAPI), Hoechst33342, Total protein extraction kit, and Total RNA Extraction Reagent were purchased from Jiangsu Keygen Biotech Corp., Ltd. (Nanjing, China). TIANamp Genomic DNA Kit was obtained from TIANGEN BIOTECH (BEIJING) CO., LTD. (Beijing, China). All ELISA Kits were obtained from MultiSciences (Lianke) Biotech Co., Ltd. (Hangzhou, China). FEN1‐IN‐4 (HY‐136485) and 8‐OH‐dG rabbit antibody (HY‐P81140) were purchased from MCE (New Jersey, USA). ABflo 647‐conjugated Goat anti‐Rabbit IgG (H+L) (AS060), IL‐1β Rabbit pAb (A16288), NLRP3 Rabbit mAb (A24294), STING/TMEM173 Rabbit mAb (A21051), FEN1 Rabbit pAb (A1175), TLR9 Rabbit pAb (A14642), SQSTM1 Rabbit mAb (A19700), NF‐κB Rabbit mAb (A19653), GAPDH Rabbit mAb (A19056) and β‐actin Rabbit mAb (AC026) were purchased from ABclonal Technology (Wuhan, China). Anti‐DNA antibody (AC‐30‐10) was from Sigma–Aldrich (USA). PE/Cyanine7 anti‐mouse Ly‐6G (127617), APC anti‐mouse F4/80 (123115), and purified anti‐mouse CD86 (MMR) antibody (105001) were from BioLegend (California, USA). FITC‐Ep‐CAM mouse monoclonal antibody was obtained from Santa Cruz Biotechnology, Inc. (Dallas, USA). CD206 Polyclonal antibody (18704‐1‐AP), cGAS Recombinant antibody Rabbit (84045‐1‐RR), and GPX4 monoclonal antibodies (67763‐1‐Ig) were purchased from Proteintech Group, Inc. (Wuhan, China).

### 4.2 Synthesis of FEN1 Inhibitor

The synthesis route of FEN‐inhibitor (FI) was shown in Figure S2A. Methyl anthranilate reacted with 4‐dimethylaminopyridine (DMAP) and N, N'‐carbonyldiimidazole (CDI) in toluene solution for 3 h. Then, O‐(tetrahydro‐2H‐pyran‐2‐yl) hydroxylamine was added, and the mixture was heated for another 3 h. After purification by reduced‐pressure distillation, the compound 3‐[(tetrahydro‐2H‐pyran‐2‐yl)oxy]‐2,4(1H,3H)‐quinazolinedione was obtained. Subsequently, it reacted with (4‐bromobutyl) triphenylphosphonium to introduce the TPP group into the compound, yielding the mitochondria‐targeted FEN1 inhibitor (FI).

### 4.3 Fabrication of SeNPs and Se‐FI NPs

SeNPs were synthesized by the chemical reduction method. Na_2_SeO_3_ and ascorbic acid were separately dissolved in pure water. Briefly, under stirring conditions, 5 mL of the ascorbic acid solution (200 mM) was slowly added dropwise to 50 mL of the Na_2_SeO_3_ solution (2 mM) and reacted for 15 min. Then, the aqueous solution of chitosan (MW: 5000, 10 mg/mL) was added as a stabilizer. The mixture was continuously stirred at 35°C for 6 h. After that, the liquid was collected and centrifuged at 14 000 rpm for 20 min to remove unreacted substances. Finally, the nanoparticles were washed twice with ultrapure water to obtain the desired SeNPs.

A mixed aqueous solution of chitosan and FI (Cs/FI = 1:1) was used as the reaction stabilizer, and the rest of the preparation process was the same as that for SeNPs to obtain the Se‐FI NPs.

### 4.4 Synthesis of DSPE‐PEG‐HA

DSPE‐PEG‐HA was obtained through a condensation reaction. EDC (0.0313 mM), NHS (0.0313 mM), and HA (0.0313 mM) with different molecular weights (60, 100, 200, and 400 kD) were co‐dissolved in 5 mL of formamide, followed by ultrasonication for 10 min to facilitate dissolution. The mixture was then stirred at room temperature for 1 h to activate the carboxyl groups. Subsequently, 5 mL of DMF solution containing DSPE‐PEG‐NH_2_ (0.05 mM) was added to the stirring solution. After reacting at room temperature for 24 h, the mixture was dialyzed against DMSO for 24 h and then against pure water for 48 h. Finally, freeze‐drying was performed to obtain DSPE‐PEG‐HA powder. Fourier transform infrared spectroscopy (FTIR) was performed to confirm the structures.

### 4.5 Preparation of SeNPs@Lip‐HA, Se‐FI@Lip and Se‐FI@Lip‐HA

For SeNPs@Lip‐HA formulation, PC, cholesterol, DSPE‐mPEG and DSPE‐mPEG‐HA (at a molar ratio of 75:20:2:3) were dissolved in 5 mL of dichloromethane. The organic solvent was removed by reduced‐pressure evaporation at 37°C until a homogeneous and transparent film was formed. Subsequently, 5 mL of SeNPs aqueous solution (0.4 mg/mL) was added for hydration at 45°C for 15 min. The resulting solution was subjected to ultrasonic homogenization for 5 min, yielding the nano‐formulation SeNPs@Lip‐HA.

For the Se‐FI@Lip formulation, the lipid formulation consisted of PC, cholesterol, and DSPE‐mPEG (at a molar ratio of 75:20:5), and the hydration solvent was replaced with the Se‐FI NPs aqueous solution (0.4 mg/mL), finally yielding the HA‐unmodified liposomes.

For the Se‐FI@Lip‐HA formulation, the lipid formulation consisted of DSPE‐mPEG and DSPE‐mPEG‐HA (at a mol ratio of 75:20:2:3), and the hydration solvent was the Se‐FI NPs aqueous solution (0.4 mg/mL). Fluorescently labeled nanoparticles were prepared by the same method, with Ce6 or DiI dissolved in dichloromethane together with lipids.

### 4.6 Characterization of the Prepared Nanoparticles

The particle size distributions, PDI, zeta potential, and formulation stability were measured by dynamic light scattering (DLS) (Zeta Plus, Brookhaven, USA). Morphological characteristics and the elemental distribution on the surface and inside of the nanoparticles were determined via transmission electron microscopy (TEM) (JEOL JEM‐F200, Japan), mapping, and the elemental valence state was investigated by x‐ray photoelectron spectroscopy (XPS) (Thermo Scientific K‐Alpha, USA). The EE and DL of FI were calculated through High‐performance liquid chromatography (HPLC) (Agilent 1220 Infinity II, USA).

### 4.7 Antioxidant Capacity of SeNPs

The ·OH scavenging activity of SeNPs was determined by the TMB chromogenic method. Specifically, 1.148 g of sodium acetate was dissolved in 50 mL of pure water, followed by the addition of 0.914 mL of glacial acetic acid to prepare HAc/NaAc buffer solution (0.5 M, pH 4.5). Then, 2.4 mg of 3,3',5,5'‐tetramethylbenzidine (TMB) was dissolved in 2 mL of DMSO, and the resulting solution was diluted to 0.5 mM using the prepared HAc/NaAc buffer. Subsequently, the 4 mM H_2_O_2_ solution and 2 mM FeSO_4_ solution were mixed (v/v, 1/1) and reacted for 10 min. After that, SeNPs solutions with different concentrations (6.25, 12.5, 25, 50, 100, 200, 400 µg/mL) and the prepared TMB solution were co‐incubated for 30 min. Finally, the absorbance at 652 nm was measured to evaluate the ·OH scavenging capacity of SeNPs at different concentrations.

For the determination of total antioxidant capacity, the ABTS stock solution (7 mM) was mixed and followed by a reaction at room temperature in the dark for 16 h to obtain the ABTS working stock solution. The stock solution was diluted with PBS to adjust its absorbance at 734 nm to 0.7 ± 0.05. Subsequently, the diluted ABTS solution was incubated with SeNPs for 5 min, and the absorbance at 734 nm was measured to evaluate the total antioxidant capacity of SeNPs at different concentrations (25, 50, 100, 200, 400, 500 µg/mL).

The DPPH radical scavenging capacity and ·O_2_^−^ scavenging capacity of SeNPs at different concentrations (31.25, 62.5, 125, 250, 500, 750, 1000 µg/mL) were evaluated using the DPPH Radical Scavenging Capacity Assay Kit and the Superoxide Anion Radical Assay Kit, respectively.

### 4.8 ESR Analysis

Electron Spin Resonance (ESR) (Bruker EMXplus‐6/1, Germany) was employed to determine the free radical content. The ·OH and ·O_2_^−^ radical systems were each co‐incubated with SeNPs for 10 min. The scavenging effects of SeNPs and Se‐FI NPs on ·OH and ·O_2_^−^ were evaluated by observing the changes in the peak intensity of ESR spectra.

### 4.9 Cell Culture

RAW264.7 and 16HBE cells were purchased from ATCC (Shanghai, China); MLE‐12 cells were obtained from iCell Bioscience Inc. (Shanghai, China). RAW264.7 was cultured in RPMI‐1640 medium containing 10% FBS, 16HBE cells were cultured in DMEM medium with 10% FBS, and MLE‐12 cells were cultured in DMEM/F12 medium with 10% FBS. All cells were incubated in the incubator at 37°C with 5% CO_2_.

### 4.10 Cell Viability Assay

RAW264.7 cells, 16‐HBE cells, and MLE‐12 cells were seeded into 96‐well plates and incubated overnight for adherence. After discarding the culture medium, the cells were co‐incubated with different concentrations of Se‐FI@Lip‐HA (10, 20, 30, 40, 50, 60, 70, 80, 100 µg/mL) for 24 or 48 h, respectively. Subsequently, the medium was aspirated, and 100 µL of serum‐free medium containing MTT reagent was added to each well.

RAW264.7 cells were seeded in 6‐well plates and polarized into pro‐inflammatory M1 macrophages by induction with LPS solution (2.5 µg/mL). Subsequently, the cells were incubated with Se‐FI@Lip‐HA solutions of different concentrations (12.5, 25, 50, 100 µg/mL) for 24 h. Then, dual‐fluorescence staining of live and dead cells was performed using Calcein AM and PI in combination. The fluorescence intensities of the two fluorophores were observed under an inverted fluorescence microscope (Olympus IX53, Japan), and the cell viability and cytotoxicity were evaluated based on the corresponding fluorescence intensities.

### 4.11 Evaluation of the Cytotoxicity of FI and FEN1‐IN‐4

MLE‐12 cells were seeded into 96‐well plates and stimulated by LPS; then the cells were separately incubated with FI and FEN1‐IN‐4 solutions of different concentrations for 12, 24, and 48 h. The culture medium was then aspirated, and 100 µL of MTT reagent (0.5 mg/mL) was added to each well. After incubation for 4 h, the absorbance at 570 nm was measured.

### 4.12 Screening of HA With Optimal Molecular Weight

To evaluate the targeting effect of HA modification, the cellular uptake rates of NPs grafted with HA of different molecular weights were investigated. RAW264.7 cells were seeded in confocal laser dishes (1 × 10^5^ cells per well) and polarized into M1. Subsequently, the cells were incubated with Ce6‐labeled Se‐FI@Lip‐HA_60_, Se‐FI@Lip‐HA_100_, Se‐FI@Lip‐HA_200_, and Se‐FI@Lip‐HA_400_ for 2, 4, and 8 h, respectively. After three washes with PBS, the cells were fixed for 15 min with 4% paraformaldehyde and stained with DAPI. After washing with PBS, the cellular uptake of NPs was detected by confocal laser microscope (CLSM) (Zeiss LSM800, Germany) and flow cytometry.

### 4.13 Cell Uptake

RAW264.7 cells were seeded in confocal laser dishes (1 × 10^5^ cells per well) and polarized into M1. Subsequently, cells were treated respectively with Ce6‐labeled Se‐FI@Lip and Se‐FI@Lip‐HA (Ce6: 0.4 µg/mL) for 2, 4, and 8 h. After three washes with PBS, the cells were fixed for 15 min with 4% paraformaldehyde and stained with DAPI. After washing with PBS, the cellular uptake of NPs was observed and photographed using the CLSM.

MLE‐12 cells were seeded in confocal laser dishes (1 × 10^5^ cells per well) and treated respectively with different concentrations of Ce6‐labeled Se‐FI@Lip and Se‐FI@Lip‐HA (Ce6: 1, 2, 3 µg/mL) for 4 h. After the cells were rinsed with PBS and stained with DAPI, cell uptake was detected by CLSM.

### 4.14 Intracellular ROS Detection

RAW264.7 cells and MLE‐12 cells were uniformly seeded in confocal laser dishes (1 × 10^5^ cells per well) and 6‐well plates (2.5 × 10^5^ cells per well), then cells were stimulated with LPS. After that, the cells were separately incubated with PBS, SeNPs, SeNPs@Lip‐HA, Se‐FI@Lip, and Se‐FI@Lip‐HA, or different concentrations of Se‐FI@Lip‐HA (12.5, 25, 50, 100 µg/mL) for 24 h, followed by incubation with DCFH‐DA solution at 37°C for 30 min. Subsequently, the fluorescence intensity was observed and recorded using CLSM and flow cytometer, respectively.

### 4.15 Measurement of Mitochondrial ROS

RAW264.7 cells and MLE‐12 cells were seeded in confocal laser dishes and 6‐well plates overnight and then induced by LPS (2.5 µg/mL). Subsequently, the cells were treated with PBS, SeNPs, SeNPs@Lip‐HA, Se‐FI@Lip, and Se‐FI@Lip‐HA for 24 h, followed by incubation with MitoSOX at 37°C for 30 min. Following the procedure described by Wang et al. [45]. The cells were then incubated with MitoTracker Green for another 30 min to stain the mitochondria. The cells were rinsed with PBS, and then subjected to a 30‐min incubation with Hoechst 33342 or DAPI. Finally, the MFI was quantified by CLSM and flow cytometer.

### 4.16 Anti‐Inflammatory and Antioxidant Effect Analysis In Vitro

RAW264.7 cells were inoculated in 6‐well plates overnight and then induced into M1 macrophages. Subsequently, the cells were separately incubated with PBS, SeNPs, SeNPs@Lip‐HA, Se‐FI@Lip, and Se‐FI@Lip‐HA, or different concentrations of Se‐FI@Lip‐HA (12.5, 25, 50, 100 µg/mL) for 24 h. Then, the culture medium was collected. Following the protocols of manufacturers, the cytokines and oxidative stress indexes were measured using enzyme‐linked immunosorbent assay (ELISA) kits. The cytokines tested included mouse TNF‐α, IL‐1β, IL‐6, and CCL2; the oxidative stress indexes included MDA, GPX, and GSSG/GSH.

MLE‐12 cells were inoculated in 6‐well plates overnight and treated with 2.5 µg/mL of LPS, and then the cells were separately incubated with PBS, SeNPs, SeNPs@Lip‐HA, Se‐FI@Lip and Se‐FI@Lip‐HA for 24 h. Subsequently, the cell supernatant was collected to determine the content of TNF‐α, IL‐1β, IL‐6, and CCL2.

### 4.17 Detection of Epithelial Cell Survival

RAW264.7 cells were seeded in 6‐well plates and cultured overnight. The experimental groups were set as follows: Control group, PBS group, SeNPs group, SeNPs@Lip‐HA group, Se‐FI@Lip group, and Se‐FI@Lip‐HA group. The cells were induced to polarize into M1 macrophages using LPS (2.5 µg/mL), while the Control group was left untreated. After 8 h of induction, the cells were transferred to the upper chambers of Transwell inserts. Meanwhile, MLE‐12 cells were seeded in the lower chambers of the Transwell plates and allowed to adhere for 12 h. Subsequently, the cells in the upper chambers were treated with SeNPs, SeNPs@Lip‐HA, Se‐FI@Lip, and Se‐FI@Lip‐HA, respectively, with no drug administration applied to the PBS group. The two types of cells were further co‐incubated for 24 h. After that, the MLE‐12 cells in the lower chambers were subjected to dual‐fluorescence staining with Calcein AM and PI, respectively. The fluorescence intensities of the two dyes were observed under the inverted fluorescence microscope.

### 4.18 Observation of Macrophage Reprogramming

RAW264.7 cells were inoculated in laser confocal dishes overnight and then induced into M1 macrophages as previously reported [58]. Subsequently, the cells were separately incubated with PBS, SeNPs, SeNPs@Lip‐HA, Se‐FI@Lip, and Se‐FI@Lip‐HA for 24 h. Then, the cells were fixed with 4% paraformaldehyde solution for 15 min, followed by permeabilization with 0.1% Triton X‐100. After washing with PBS for 3 times, the cells were blocked with 1% bovine serum albumin (BSA) at room temperature for 30 min. Then, the cells were incubated with mouse anti‐CD86 and rabbit anti‐CD206 primary antibodies overnight at 4°C. After that, the cells were incubated with Alexa Fluor 647‐ conjugated goat anti‐rabbit IgG and Alexa Fluor 488‐conjugated goat anti‐mouse IgG secondary antibodies at room temperature for 2 h, followed by DAPI staining. Finally, the fluorescence intensity was observed and photographed by CLSM.

### 4.19 Immunofluorescence

For the detection of oxidized mitochondrial DNA (ox‐mtDNA) and mitochondria, RAW264.7 cells were placed in confocal dishes overnight and then induced into M1 macrophages. Subsequently, the cells were separately incubated with PBS, SeNPs, SeNPs@Lip‐HA, Se‐FI@Lip, and Se‐FI@Lip‐HA for 24 h. After treatment, the cells were incubated with MitoTracker Green at 37°C for 20 min and fixed with 4% paraformaldehyde solution for 15 min, and permeabilized with 0.1% Triton X‐100 for 10 min at room temperature. Subsequently, the cells were blocked with 1% BSA at room temperature for 30 min. After washing with PBS, the cells were incubated with 8‐OH‐dG Rabbit Antibody (1:400, MCE) overnight at 4°C, followed by reaction with ABflo 647‐conjugated goat anti‐rabbit IgG (1:500) for 2 h at room temperature and stained with DAPI.

For DNA and mitochondria detection, RAW264.7 cells were inoculated in confocal dishes overnight and then induced into M1 macrophages. After treatment with formulations, the cells were incubated with PicoGreen for 45 min and MitoTracker for 30 min at 37°C. Alternatively, the cells were subjected to fixation, permeabilization, and blocking following the aforementioned protocol, then incubated with the primary anti‐DNA antibody overnight at 4°C, and subsequently incubated with ABflo 647‐conjugated goat anti‐rabbit IgG (1:200) for 2 h at 37°C. Images of the cells were captured using polar SIM.

### 4.20 Detection of Cytoplasmic mtDNA

RAW264.7 cells and MLE‐12 cells were seeded in large culture dishes and cultured overnight. The cells were then stimulated with LPS. After 8 h, the cells were treated with the corresponding formulations in the respective dishes. After 24 h, the cells were collected and resuspended in mitochondrial isolation reagent (C3601, Beyotime). Following appropriate homogenization to disrupt the cells, the homogenate was centrifuged at 1000 × *g* for 10 min, and the supernatant was transferred and further centrifuged at 12 000 × *g* for 10 min. The resulting supernatant was used to extract cytoplasmic DNA using the TIANamp Genomic DNA Kit (DP304, TIANGEN). Subsequently, the extracted DNA was mixed with different primers for amplification, and quantitative analysis of mitochondrial‐related genes was performed via PCR or qPCR. The primers for 18S were: Forward 5’TAGAGGGACAAGTGGCGTTC, reverse 5’CGCTGAGCCAGTCAGTGT. The primers for ND1 were: Forward 5’CGGGCTACTACAACCCTTCG, reverse 5’GCGATGGTGAGAGCTAAGGT. The primers for ND2 were: Forward 5’CTATCACCCTATTAACCACTCA, reverse 5’TTCGCCTGTAATATTGAACGTA. The primers for ND4 were: Forward 5’CAGCCACATAGCCCTCGTAG, reverse 5’CCCGTGGGCGATTATGAGAA. The primers for D‐loop were: Forward 5’AATCTACCATCCTCCGTGAAACC, reverse 5’TCAGTTTAGCTACCCCCAAGTTTAA. The relative expression levels of mitochondrial‐associated genes were normalized to 18S as an internal control and calculated via the 2^−ΔΔCT^ method.

### 4.21 RNA Isolation and RT‐qPCR Analysis

RAW264.7 cells and MLE‐12 cells were seeded in 6‐well plates and cultured overnight. After model establishment and NPs treatment, total RNA was extracted using Trizol solution (KGF5101‐100, KeyGEN). Subsequently, total RNA was reverse‐transcribed into cDNA using 5 × RT SuperMix and 8 × gDNA remover, followed by amplification with BlasTaq 2 × qPCR Master Mix kit. Relative mRNA expression levels were normalized to GAPDH as the internal reference and calculated via the 2^−ΔΔCT^ method.

### 4.22 Western Blot Assay

Cells were seeded in 6‐well plates, cultured overnight, and then treated with different formulations. Total protein was extracted, and the protein concentration was analyzed with a BCA Protein Assay Kit. After denaturation, the proteins were separated via sodium dodecyl sulfate‐polyacrylamide gel electrophoresis (SDS‐PAGE) and subsequently transferred onto a nitrocellulose (NC) membrane. The membranes were then blocked with 5% non‐fat milk and incubated with primary antibodies overnight at 4°C. Following three rounds of TBST buffer washing, the membranes were incubated with secondary antibodies for 2 h at room temperature. Subsequently, after repeating the TBST washing step three times, the blots were developed via the electrochemiluminescence (ECL) detection substrate (180–5001; Tanon), with band visualization accomplished using a CCD imaging system (Tanon 4200, Shanghai, China). Quantitative analysis of the corresponding protein expression levels was performed with ImageJ software.

### 4.23 Measurement of the Mitochondrial Membrane Potential

RAW264.7 cells were inoculated in confocal dishes overnight and then induced into M1 macrophages. After treatment with different formulations for 24 h, these cells were incubated with 1× JC‐1 staining solution for 30 min at 37°C as previously described [54]. Finally, the fluorescence intensity was observed and photographed by STELLARIS 5 (Leica, Germany).

### 4.24 Animals

BALB/c mice (male, 6–8 weeks old, and 20 ± 2 g) were provided by Jiangsu Huachuang Xinnuo Medical Technology Co., Ltd (Nanjing, China). The experimental animals were housed under standard laboratory conditions at a constant temperature of 25°C and a relative humidity of 50%, with free access to a standard rodent chow and autoclaved water. All animal experimental procedures were carried out in compliance with protocols approved by the animal ethics committee of China Pharmaceutical University (Approval No. YSL‐202507012).

### 4.25 Model Establishment and NPs Treatment

A mouse model of acute respiratory distress syndrome (ARDS) was established via intratracheal instillation of LPS (7.5 mg/kg). After 2 h, the mice were randomly assigned to five experimental groups: the PBS group, SeNPs group, SeNPs@Lip‐HA group, Se‐FI@Lip group, and Se‐FI@Lip‐HA group. Meanwhile, healthy mice without ARDS modeling were set as the control group. Different NPs were administered to the corresponding groups via intratracheal instillation, while no treatment was given to the control group. After 24 h of treatment, the mice were euthanized, and bronchoalveolar lavage fluid (BALF) was collected via lung lavage, followed by dissection to harvest lung tissues and serum for subsequent analyses. To establish a mouse model of sepsis, LPS (15 mg/kg) was administered to mice via intraperitoneal injection.

### 4.26 RNA Sequencing Data Analysis

The RNA sequencing of cells was performed by Wuhan Benna Biotechnology Co., Ltd. (Wuhan, China). The RNA sequencing data analysis was filtered by FastQC for valid reads and quality control. Gene Ontology (GO) and Kyoto Encyclopedia of Genes and Genomes (KEGG) enrichment analyses were performed with clusterProfiler.

### 4.27 Biodistribution of NPs In Vivo

After model establishment, different DiI‐labeled NPs were administered to the mice via intratracheal instillation. At 24 h post‐administration, the mice were euthanized, and the heart, liver, spleen, lung, and kidney were harvested. These tissues were then imaged using the IVIS imaging system (Tanon, China), and the distribution and retention of NPs were evaluated based on the fluorescence intensity. Meanwhile, to further assess the NPs uptake by pulmonary macrophages, neutrophils, and epithelial cells, the dissected lung tissues were immediately placed on dry ice. Following tissue fixation and sectioning, immunofluorescence staining was performed with F4/80, Ly6G, and EpCAM markers, and subsequent colocalization analysis was carried out.

After establishing the model and administering different DiI‐labeled NPs for 24 h, mice lung tissue was collected and digested to obtain lung single‐cell suspensions. RBCs were lysed, and the samples were centrifuged at 2000 rpm for 10 min. The resulting cells were collected and incubated with fluorescent primary antibody for 2 h. The fluorescence intensity of each marker was then analyzed by flow cytometry.

### 4.28 Histological and Immunohistochemical (IHC) Analysis

After harvesting the lung tissues, frozen sections were prepared and subjected to DHE staining for the detection of ROS levels in the lung tissues. The major organs were excised and immersed in 4% paraformaldehyde for fixation, followed by the preparation of paraffin‐embedded sections for hematoxylin‐eosin (H&E) staining, and IHC staining.

### 4.29 BALF Analysis

BALF was collected by lavaging the lungs three times with cold normal saline. After erythrocyte lysis, the total protein concentration in BALF was quantified using the BCA assay. Total cell counts were determined with a hemocytometer. Total DNA was extracted from BALF for PCR and qPCR analyses to assess nucleic acid levels. Additionally, ELISA kits were employed to measure the concentrations of inflammatory cytokines and oxidized DNA.

### 4.30 Pulmonary Permeability Analysis

Lung barrier permeability was assessed via the Evans Blue assay. Evans Blue dye (30 mg/kg, dissolved in normal saline) was administered to mice via tail vein injection 2 h before euthanasia. Subsequently, 3 mL of PBS was injected into the right ventricle to flush out residual dye from the pulmonary blood vessels. The lung tissues were harvested, homogenized in formamide, and incubated at 60°C for 24 h. Following centrifugation, the absorbance of the supernatant was measured at 620 nm, and the extent of lung barrier disruption was evaluated based on the optical density (OD) values.

### 4.31 Wet/Dry Ratios of Lungs

The mice were euthanized and dissected to harvest the lung tissues, which were immediately weighed to obtain the wet weight. Subsequently, the lung tissues were placed in an oven at 65°C for 72 h for drying, and then weighed again to determine the dry weight. The degree of pulmonary edema was evaluated by calculating the ratio of wet weight to dry weight (W/D ratio).

### 4.32 Pulmonary Function Test

Respiratory function parameters of mice were detected using a whole‐body plethysmography system (WBP, ELM204, UK). The mice were placed in the detection chamber, and after their breathing stabilized, the tidal volume (TV), pause enhanced (Penh), minute volume (MV), and relaxation time (tR) were measured.

### 4.33 Safety Assessment

Major tissues were harvested, fixed, and embedded in paraffin, followed by sectioning for hematoxylin‐eosin (H&E) staining to evaluate organ damage. A portion of the blood sample was subjected to a complete blood count (CBC) analysis, whereas the remaining portion was allowed to stand at room temperature for 2 h, then centrifuged at 3000 × *g* for 10 min to prepare serum. The concentrations of alanine transaminase (ALT), aspartate transaminase (AST), and blood urea nitrogen (BUN) in the serum were determined subsequently.

### 4.34 Statistical Analysis

All data are presented as mean ± standard deviation (SD) (n ≥ 3) and were analyzed using FlowJo 10, ImageJ, GraphPad Prism 8, and Origin 2021 software. One‐way analysis of variance (ANOVA) and two‐way ANOVA were used for multi‐group comparisons, followed by two‐tailed Tukey's multiple comparisons test. Raw data did not undergo preprocessing. All statistical analyses were two‐tailed, and statistical significance was defined as *p < 0.05, **p < 0.01, and ***p < 0.001, ****p < 0.0001; *p* ≥ 0.05 was considered not significant. Sample or experiment size (n) is indicated in each figure legend.

## Author Contributions

**Wen‐Ling Li**: conceptualization, writing – original draft, software, visualization, investigation. **Juan Cao**: writing – original draft, software, data curation, formal analysis. **Yu‐Lin Wang**: writing – original draft, methodology, conceptualization. **Yu‐Han Fan**: investigation, validation. **Jia‐Qi Xu**: investigation. **Xian Wu Cheng**: funding acquisition, supervision. **Lei Xing**: writing – review and editing, supervision, funding acquisition. **Hu‐Lin Jiang**: supervision, writing – review and editing, resources, project administration, funding acquisition.

## Funding

This work was financially supported by the National Natural Science Foundation of China (82020108029, 82473867). This work was also supported by Lingang Laboratory (LGL‐2611‐10), Natural Science Foundation of Jiangsu Basic Research Program‐Project jointly funded by province and city (BK20232035), the Jilin Provincial Foundation of Changbai Talent Outstanding Team (20240717129), and 2024 Project on Comprehensive Research of Multi‐Target Natural Medicines (SKLNMZZ2024JS08).

## Conflicts of Interest

The authors declare no conflicts of interest.

## Supporting information




**Supporting File 1**: advs77577‐sup‐0001‐SuppMat.docx.

**Supporting File 2**: advs77577‐sup‐0002‐DataS1.xlsx.

## Data Availability

The data that support the findings of this study are available from the corresponding author upon reasonable request.
