# Supplementary material for: Mitochondria‐Targeted Multimodal Nanotherapeutics Suppress Oxidized mtDNA‐Driven Inflammation at the Source
Source: Adv Sci (Weinh). 2026 Sep 1:e77577. Online ahead of print. doi: 10.1002/advs.77577 (PMC13534779; doi:10.1002/advs.77577)
Supplement: Supplementary file 1 [file ADVS-9999-e77577-s002.docx]

**Supporting Information**

**Mitochondria-Targeted Multimodal Nanotherapeutics Suppress Oxidized mtDNA-Driven Inflammation at the Source**

*Wen-Ling Li, Juan Cao, Yu-Lin Wang, Yu-Han Fan, Jia-Qi Xu, Xian Wu Cheng^*^, Lei Xing^*^, Hu-Lin Jiang^*^*

W.-L. Li, J. Cao, Y.-L. Wang, Y.-H. Fan, J.-Q. Xu, L. Xing, H.-L. Jiang

State Key Laboratory of Natural Medicines, Department of Pharmaceutics, China Pharmaceutical University, Nanjing, China.

E-mail: xinglei6xl@163.com E-mail: jianghulin3@163.com

X. W. Cheng, H.-L. Jiang

Jilin Provincial Key Laboratory of Stress and Cardiovascular Disease, Department of Cardiology and Hypertension, The Affiliated Hospital of Yanbian University (Yanbian University Hospital), Yanji 133000, China.

E-mail: chengxw@ybu.edu.cn E-mail: jianghulin3@163.com

H.-L. Jiang

Joint International Research Laboratory of Target Discovery and New Drug Innovation, MOE, China Pharmaceutical University, Nanjing, China.

E-mail: jianghulin3@163.com

H.-L. Jiang

Department of Precision Medicine, School of Medicine, Sungkyunkwan University, Suwon, Korea.

E-mail: jianghulin3@163.com

*Corresponding author. Email: jianghulin3@163.com or jianghulin3@cpu.edu.cn

These authors contributed equally: Wen-Ling Li, Juan Cao, and Yu-Lin Wang

# **Supplementary figures**

**Supplementary Figure 1.** Representative GSEA plots of inflammatory pathways based on GO.

**Supplementary Figure 2. A** Synthetic route of FI. **B** ^1^H NMR spectra of FI in DMSO-d6 (400 MHz). **C** LC-MS (495 nm) chromatograms of FI. **D** Schematic illustration of the two magnesium ions' metal-coordination spheres in the active site.

**Supplementary Figure 3.** **A** Association rate (Ka), dissociation rate (Kd) and dissociation constant (K_D_) of FI with FEN1 recombinant protein measured by SPR.

**B** Affinity of inhibitor FI for FEN1 assessed by isothermal titration calorimetry (ITC).

**Supplementary Figure 4.** Fluorescence microscopy images showing the live/dead status of MLE-12 treated with different concentrations of FEN1-IN-4 or FEN1-IN-4-TPP for 24 h. Live cells were stained with Calcein AM (green), and dead cells were stained with PI (red). Scale bar: 400 μm.

**Supplementary Figure 5.** **A-B** Particle size and PDI of SeNPs synthesized with chitosan of molecular weights 5000 and 10000 as stabilizers at various reaction times. The optimal molecular weight of Cs and stirring time were 5000 Da and 6 h, respectively (n = 3). **C** Photographs of Se-FI NPs solutions. Data are presented as mean ± SD.

**Supplementary Figure 6.** FI release curves at different times. Data are presented as mean ± SD.

**Supplementary Figure 7.** A-C Changes in particle size and PDI of Se-FI@Lip-HA over 7 days in PBS, saline, and medium containing 10% serum (n = 3). Data are presented as mean ± SD.

**Supplementary Figure 8. A-C** Scavenging activity of SeNPs at different concentrations against ·O_2_^-^, ·OH, and 2,2-diphenyl-1-picrylhydrazyl (DPPH) radicals.

**Supplementary Figure 9. A-D** Comparison of free radical scavenging activities of SeNPs, V_C_, and V_E_ at different concentrations against ABTS, ·OH, H_2_O_2_, and DPPH radicals (n = 3). Data are presented as mean ± SD.

**Supplementary Figure 10.** **A** Standard curve. **B** Drug loading (DL%) capacity of Ce6-labeled liposomes grafted with different molecular weight HA. Data are presented as mean ± SD.

**Supplementary Figure 11.** **A** Cell viability of 16HBE cells treated with different concentrations of Se-FI@Lip-HA for 24 or 48 hours, determined by the MTT assay (n = 4). **B** Cell viability of MLE-12 cells treated with different concentrations of Se-FI@Lip-HA for 24 or 48 hours (n = 4). **C** Cell viability of RAW264.7 cells treated with different concentrations of Se-FI@Lip-HA for 24 or 48 hours (n = 4). **D** Fluorescence microscopy images showing the live/dead status of inflammatory macrophages treated with different concentrations of Se-FI@Lip-HA for 24 hours. Live cells were stained with Calcein AM (green), and dead cells were stained with PI (red). Scale bar: 100 μm. Data are presented as mean ± SD.

**Supplementary Figure 12.** **A** Drug loading (DL%) capacity of Ce6 in Ce6-labeled Se-FI@Lip and Se-FI@Lip-HA nanoparticles (n = 3). **B** Quantitative analysis of fluorescence intensity from CLSM images showing cellular uptake at different time points. Data are presented as mean ± SD.

**Supplementary Figure 13.**  Live/dead staining images of MLE-12 cells after co-culture with inflammatory macrophages and subsequent treatment with different groups. Live cells were stained with Calcein AM (green), and dead cells were stained with PI (red). Scale bar: 100 μm.

**Supplementary Figure 14.** **A** Confocal microscopy images showing intracellular ROS scavenging by different concentrations of Se-FI@Lip-HA in LPS-stimulated RAW264.7 macrophages. DCFH-DA served as the ROS probe (green). Scale bar: 20 μm. **B** Quantitative analysis of DCFH-DA fluorescence intensity from confocal microscopy images. **C** Flow cytometry analysis of intracellular ROS scavenging by various concentrations of Se-FI@Lip-HA in LPS-stimulated RAW264.7 macrophages.

**Supplementary Figure 15.** Flow cytometry analysis of mtROS in RAW264.7 cells treated with different formulations after LPS stimulation.

**Supplementary Figure 16.** Mitochondrial Se content detected by ICP-MS (n = 3). Data are presented as mean ± SD and analyzed by one-way ANOVA with Tukey’s multiple comparisons test. **p* < 0.05, ***p* < 0.01, and ****p* < 0.001, *****p* < 0.0001.

**Supplementary Figure 17.** MDA and GPX levels, and GSSG/GSH ratio in inflamed RAW264.7 cell culture medium after treatment with different concentrations of Se-FI@Lip-HA (n = 3). Data are presented as mean ± SD and analyzed by one-way ANOVA with Tukey’s multiple comparisons test. **p* < 0.05, ***p* < 0.01, and ****p* < 0.001, *****p* < 0.0001.

**Supplementary Figure 18.** Colocalization determined by the selected white line across the entire cell in Polar-SIM analysis.

**Supplementary Figure 19.** Nucleic acid gel electrophoresis of cytosolic mtDNA extracted from macrophages after LPS stimulation and treatment with SeNPs@Lip-HA or Se-FI@Lip-HA. MtDNA fragments less than 750 bp in each group are shown in red box.

**Supplementary Figure 20.** Determination of extramitochondrial mtDNA content in inflamed RAW264.7 cells treated with different formulations via qPCR using ND2 and D-loop primers (n = 3). Data are presented as mean ± SD and analyzed by one-way ANOVA with Tukey’s multiple comparisons test. **p* < 0.05, ***p* < 0.01, and ****p* < 0.001, *****p <* 0.0001.

**Supplementary Figure 21.** Quantitative data of MMP detection (n = 3). Data are presented as mean ± SD and analyzed by one-way ANOVA with Tukey’s multiple comparisons test. **p* < 0.05, ***p* < 0.01, and ****p* < 0.001, *****p* < 0.0001.

**Supplementary Figure 22**. WB assay of autophagy-related protein levels after chloroquine (CQ) co-treatment (n = 3). Data are presented as mean ± SD and analyzed by one-way ANOVA with Tukey’s multiple comparisons test. **p* < 0.05, ***p* < 0.01, and ****p* < 0.001, *****p* < 0.0001.

**Supplementary Figure 23.** Levels of TNF-α, IL-1β, IL-6 and CCL2 in the culture medium of inflamed RAW264.7 cells after treatment with different concentrations of Se-FI@Lip-HA (n = 3). Data are presented as mean ± SD and analyzed by one-way ANOVA with Tukey’s multiple comparisons test. **p* < 0.05, ***p* < 0.01, and ****p* < 0.001, *****p* < 0.0001.

**Supplementary Figure 24.** Quantitative analysis of NLRP3 and cGAS expression in MLE-12 cells treated with different experimental groups (n = 3). Data are presented as mean ± SD and analyzed by one-way ANOVA with Tukey’s multiple comparisons test. **p* < 0.05, ***p* < 0.01, and ****p* < 0.001, *****p* < 0.0001.

**Supplementary Figure 25.** cGAMP concentration and caspase-1 activity in LPS-primed macrophages after different treatments (n = 3). Data are presented as mean ± SD and analyzed by one-way ANOVA with Tukey’s multiple comparisons test. **p* < 0.05, ***p* < 0.01, and ****p* < 0.001, *****p* < 0.0001.

**Supplementary Figure 26. A, B** WB assay and quantification of TLR9 expression in RAW264.7 cells treated with different formulations after stimulation with mtDNA (n = 3). **C** The levels of TNF-α and IL-6 in RAW264.7 cells treated with different formulations after stimulation with mtDNA (n = 3). Data are presented as mean ± SD and analyzed by one-way ANOVA with Tukey’s multiple comparisons test. **p* < 0.05, ***p* < 0.01, and ****p* < 0.001, *****p* < 0.0001.

**Supplementary Figure 27. A** WB assay and quantification of TLR9 expression in MLE-12 cells treated with different formulations after stimulation with mtDNA (n = 3). **B** Levels of IL-6, TNF-α, and CCL2 in the culture medium of MLE-12 cells treated with different formulations after stimulation with mtDNA (n = 3). Data are presented as mean ± SD and analyzed by one-way ANOVA with Tukey’s multiple comparisons test. **p* < 0.05, ***p* < 0.01, and ****p* < 0.001, *****p* < 0.0001.

**Supplementary Figure 28.** CLSM images of CD86 (M1 marker, green) and CD206 (M2 marker, red) on pre-stimulated RAW264.7 after treated with different formulations. Scale bar: 20 μm.

**Supplementary Figure 29. A** Fluorescence images of mouse organs at 24 h after pulmonary administration of DiI-labeled SeNPs@Lip-HA, Se-FI@Lip and Se-FI@Lip-HA. **B** Fluorescence images of the five lobes of mouse lungs at 24 h after pulmonary administration of DiI-labeled SeNPs@Lip-HA, Se-FI@Lip and Se-FI@Lip-HA.

**Supplementary Figure 30.** H&E images of whole lung tissues from different treatment groups. Scale bar: 2000 μm.

**Supplementary Figure 31. A** The number of total cells in BALF (n = 5). **B** Quantification of mtDNA content in BALF via qPCR using ND1 and ND4 primers (n = 3). Data are presented as mean ± SD and analyzed by one-way ANOVA with Tukey’s multiple comparisons test. **p* < 0.05, ***p* < 0.01, and ****p* < 0.001, *****p* < 0.0001.

**Supplementary Figure 32.** Measurement of lung function indicators, including pause enhanced (Penh), respiratory rate (Rpef) and relaxation time (tR) (n = 5). Data are presented as mean ± SD and analyzed by one-way ANOVA with Tukey’s multiple comparisons test. **p* < 0.05, ***p* < 0.01, and ****p* < 0.001, *****p* < 0.0001.

**Supplementary Figure 33. A** Representative immunofluorescence images of the lung tissues from ARDS model mice stained with SQSTM1 (green), GPX4 (red) and DHE (red) in different treatment groups. Scale bar: 100 μm. **B** SOD and MPO levels in mouse lung homogenate (n = 5). Data are presented as mean ± SD and analyzed by one-way ANOVA with Tukey’s multiple comparisons test. **p* < 0.05, ***p* < 0.01, and ****p* < 0.001, *****p* < 0.0001.

**Supplementary Figure 34.** Levels of TNF-α, IL-18 and CXCL1 in BALF (n = 5). Data are presented as mean ± SD and analyzed by one-way ANOVA with Tukey’s multiple comparisons test. **p* < 0.05, ***p* < 0.01, and ****p* < 0.001, *****p* < 0.0001.

**Supplementary Figure 35. A** Flow cytometry analysis of neutrophil count in mouse BALF after treatment in different groups. **B** Giemsa-wright staining images of cells in BALF. Scale bar: 50 μm.

**Supplementary Figure 36. A-B** Flow cytometry analysis of macrophage phenotypes in mouse BALF after treatment in different groups (CD86^+^ for M1, CD206^+^ for M2). **C** Immunofluorescence images of mouse lung tissues stained with anti-CD86 (green) and anti-CD206 (red). Scale bar: 100 μm. **D** Levels of IL-10 in BALF (n = 5). Data are presented as mean ± SD and analyzed by one-way ANOVA with Tukey’s multiple comparisons test. **p* < 0.05, ***p* < 0.01, and ****p* < 0.001, *****p* < 0.0001.

**Supplementary Figure 37. A** H&E-stained images of mouse heart, liver, spleen and kidney after treatment in different groups. Scale bar: 100 μm. **B** Complete blood count (CBC) analysis of ARDS mice after treatment with different formulations (including RBC, PLT and HGB) (n = 5). **C** ALT, AST and BUN levels analysis in ARDS mouse serum after treatment with different formulations (n = 5). Data are presented as mean ± SD.

**Supplementary Figure 38.** H&E images of lung sections from sepsis-induced ARDS after treatment with different formulations, scale bars in the panels are 100 µm and 20 µm, respectively.

**Supplementary Figure 39. A** Flow cytometry analysis of neutrophil count in sepsis-induced ARDS mouse peripheral blood after treatment with different formulations. **B** Quantification of mtDNA content in BALF via qPCR using ND1 primer (n = 3). Data are presented as mean ± SD and analyzed by one-way ANOVA with Tukey’s multiple comparisons test. **p* < 0.05, ***p* < 0.01, and ****p* < 0.001, *****p* < 0.0001.

**Supplementary Figure 40. A** Levels of ALT, AST and BUN in sepsis-induced ARDS mouse serum after treatment with different formulations (n=3). **B** CBC analysis of sepsis-induced ARDS mice after treatment with different formulations (including WBC, RBC, PLT and HGB) (n=3). Data are presented as mean ± SD and analyzed by one-way ANOVA with Tukey’s multiple comparisons test. **p* < 0.05, ***p* < 0.01, and ****p* < 0.001, *****p* < 0.0001.

**Supplementary Figure 41.** Representative immunofluorescence images of the lung tissues from sepsis-induced ARDS mice after treatment with different formulations, stained with IL-1β, GPX4 and MPO. Scale bars in the panels are 100 µm and 50 µm, respectively.

**Supplementary Figure 42. A** Immunofluorescence images of the lung tissues from sepsis-induced ARDS mice after treatment with different formulations, stained with SQSTM1 (green). Scale bar: 100 μm. B Immunofluorescence images of lung tissues from sepsis-induced ARDS mice after treatment with different formulations, stained with anti-CD86 (green) and anti-CD206 (red). Scale bar: 100 μm.
